# A dynamic approach for visualizing and exploring concept hierarchies from textbooks

**DOI:** 10.3389/frai.2024.1285026

**Published:** 2024-02-08

**Authors:** Sabine Wehnert, Praneeth Chedella, Jonas Asche, Ernesto William De Luca

**Affiliations:** ^1^Faculty of Computer Science, Human-Centred Artificial Intelligence, Otto von Guericke University Magdeburg, Magdeburg, Saxony-Anhalt, Germany; ^2^Human-Centred Technologies for Educational Media, Leibniz Institute for Educational Media, Georg Eckert Institute, Brunswick, Germany; ^3^Faculty of Law, Economics and Business, Martin-Luther-University Halle-Wittenberg, Halle, Saxony-Anhalt, Germany

**Keywords:** hierarchy visualization, human-centered design, usability test, concept hierarchy, high-fidelity prototype, legal artificial intelligence

## Abstract

In this study, we propose a visualization technique to explore and visualize concept hierarchies generated from a textbook in the legal domain. Through a human-centered design process, we developed a tool that allows users to effectively navigate through and explore complex hierarchical concepts in three kinds of traversal techniques: top-down, middle-out, and bottom-up. Our concept hierarchies offer an overview over a given domain, with increasing level of detail toward the bottom of the hierarchy which is consisting of entities. In the legal use case we considered, the concepts were adapted from section headings in a legal textbook, whereas references to law or legal cases inside the textbook became entities. The design of this tool is refined following various steps such as gathering user needs, pain points of an existing visualization, prototyping, testing, and refining. The resulting interface offers users several key features such as dynamic search and filter, explorable concept nodes, and a preview of leaf nodes at every stage. A high-fidelity prototype was created to test our theory and design. To test our concept, we used the System Usability Scale as a way to measure the prototype's usability, a task-based survey to asses the tool's ability in assisting users in gathering information and interacting with the prototype, and finally mouse tracking to understand user interaction patterns. Along with this, we gathered audio and video footage of users when participating in the study. This footage also helped us in getting feedback when the survey responses required further information. The data collected provided valuable insights to set the directions for extending this study. As a result, we have accounted for varying hierarchy depths, longer text spans than only one to two words in the elements of the hierarchy, searchability, and exploration of the hierarchies. At the same time, we aimed for minimizing visual clutter and cognitive overload. We show that existing approaches are not suitable to visualize the type of data which we support with our visualization.

## 1 Introduction

Nowadays, people can gather almost any information from the internet. However, there are some domains where experts rely on textbooks, journals, and other forms of documents for research. Textbooks are still considered the primary source of information for many people to acquire knowledge. There have been advancements in technology that have changed the way people read these textbooks. Readers of growing textbook collections face a number of problems when comparing and connecting various parts of information in textbooks and similar areas, such as difficulty in understanding the big picture, difficulty in memorization, inconsistency between one book and another, difficulty in identifying key concepts, and the overall time-consuming process to find information when they would like to grasp a concept quickly. Particularly in certain domains, such as law, it may be necessary for a domain expert to know which textbook or version of the textbook to refer to for a specific purpose. This adds to the overall complexity of working with textbooks.

To this end, advancements in the field of Natural Language Processing (NLP) can help to overcome this problem. With NLP techniques, it has become easier to identify key concepts in documents and their relationships with one another, creating a big picture. This information can be further organized in the form of hierarchical graphs which are referred to as “Concept Hierarchies.” We introduce the basics of concept hierarchies in the following Section 2.1.1. There has been a lot of research in generating these hierarchies and visualizing them. Given that concept hierarchies are being used for a variety of purposes, it is difficult though to use standard visualization techniques to explore and visualize concept hierarchies in the use case of textbooks. Hence, there is a need to identify visualization techniques that are suited for the problem, or, to come up with a new visualization approach that fulfills the user requirements for the given use case.

**Figure 1:**
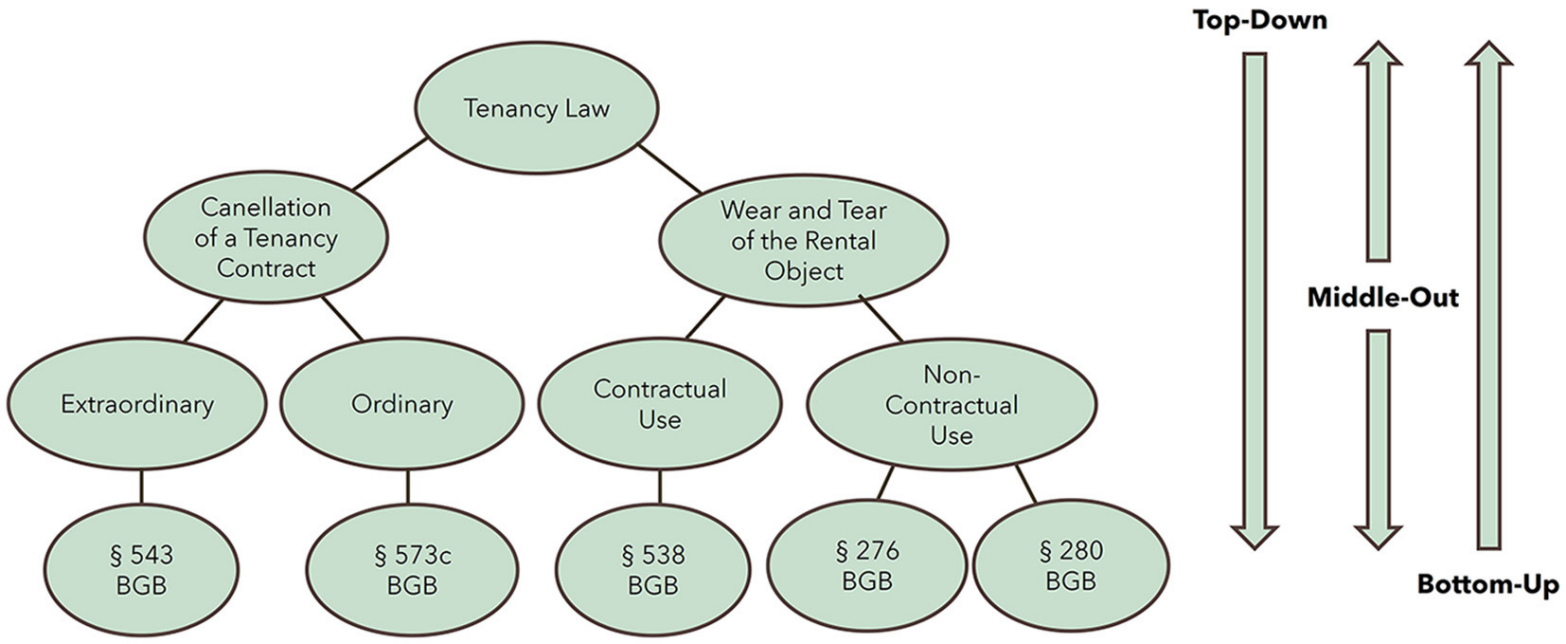
Traversal modes for the concept hierarchy: top-down, middle-out, and bottom-up.

The main goal of this study is to visualize the concept hierarchies generated from textbooks in the legal domain and to evaluate how they are perceived by users in terms of usability. We designed this visualization keeping in mind to make it as simple as possible for users/stakeholders to understand and navigate easily within hierarchies and not to lose the big picture when moving within the hierarchies. For this, we pose the following research questions based on three traversal modes within the hierarchy (see also an example of concept hierarchy in Figure 1):

**1. Which visualization is suitable for a top-down search for hierarchical data?** Searching for information starting at the highest level in a hierarchy and then drilling down further levels until the desired element is found is referred to as top-down search (Uschold and Gruninger, 1996; Guarino, 1998). This approach prevents users from information overload by letting them choose the path they would like to dive deeper into, while having an understanding of the context in which the particular node/concept is relevant in the big picture. In the context of this study, the highest level (root node) is the title of a book, and the lowest level (leaf node) is a citation/reference to law or legal cases.

**2. Which visualization is suitable for a middle-out search for hierarchical data?** Searching for information starting at a specific level in a hierarchy and then moving either to levels above or below the selected level till one reaches the information one is looking for is usually referred to as a middle-out search (Uschold and Gruninger, 1996). This approach is helpful to users when they want to explore a specific concept. In this way, they do not have to go through unwanted information by starting from the top to reach the specific concept. This approach also provides the opportunity for the user to either move up or down the hierarchy based on their interest. The highest level (root node) is the title of a book, the lowest level (leaf node) is a citation/reference, and the intermediate levels are sections/subsections/keywords.

**3. Which visualization is suitable for bottom-up search for hierarchical data?** Searching for information starting at the lowest level in a hierarchy and then moving up the levels is called bottom-up search (Uschold and Gruninger, 1996; Guarino, 1998). This approach is helpful for users who are more detail-oriented, where they start from a leaf node entity (here: a legal reference), connected to the most specific concept/topic, and learn incrementally as they move to much broader concepts/topics. Here, the lowest level (leaf node) is always a citation/reference, and the highest level (root node) is the title of a book.

Each visualization has its strengths and limitations. Since user acceptance is key, we address these questions by following human-centered design principles.

The main contributions of this study are as follows:

- We identify the need for dynamic concept hierarchy visualization methods regarding the depth of the hierarchy and the lengths of the text spans, opening the discussion in the research community about this use case.
- We use the model-based approach in human-centered design (see Section 2.3.1) for rapid prototyping based on the information we had about our user groups with expertise in the legal domain.
- To this end, we develop a high-fidelity prototype^1^ for concept hierarchy exploration and retrieval of concepts and entities, focusing on the visualization approach of the hierarchies which we extracted from a legal textbook.
- We evaluate our designed system in a usability test and share our findings.

The remainder of this study is further divided into the following parts: In Section 2, we describe the background about concept hierarchies, the related work (with a brief overview of the previous works done in the field of visualization of concept hierarchies), and the methodology for the conceptual design (model-based human-centered design approach toward a high-fidelity prototype). In Section 3, we present the results achieved for each research question. In Section 4, we conclude the study by discussing the results we obtained, further application areas, and also mention the possible future study that can be done to further improve the prototype, keeping the current limitations in mind.

## 2 Method

Before creating our own visualization method, we explored the basics of concept hierarchies. Then, we collected related work to understand design patterns and existing solutions for our use case. This is followed by the conceptual design phase, where we describe our methods for creating the high-fidelity prototype using human-centered design.

### 2.1 Background

#### 2.1.1 Definition of concept hierarchies

Concept hierarchies are a structured representation of information (concepts) and their relationships in a tree format (Büchner et al., 1998). These concept hierarchies are commonly used in areas such as artificial intelligence, informatics, and linguistics for the purpose of retrieving information or for providing a common vocabulary. A concept hierarchy mainly consists of concepts and the relationships between them. In this study, the concepts are considered as units of meaning, and the relationships between these units are considered as connections. The relationships between these concepts are usually hierarchical relationships: One concept exists either as a subtype or supertype of another concept (e.g., part-of relations).

WordNet (Miller, 1995) is a well-known concept hierarchy. It organizes words into synsets (sets of synonyms) and into a hierarchy based on their meaning. Cyc ontology (Elkan and Greiner, 1993) developed by Cycorp is a large general-purpose ontology that contains hierarchies covering various domains.

#### 2.1.2 Concept hierarchies generated from textbooks

Concept hierarchies from textbooks are typically generated through a combination of expert knowledge (Noy and McGuinness, 2001) and automated methods. There are various ways of generating concept hierarchies. The concept hierarchies used in this study are generated from legal textbooks, automatically. As a basis, we use the following workflow for extracting concept hierarchies from legal textbooks (Wehnert et al., 2018):

- The first step is to gather the textbooks in a pdf format, then convert them into txt format for further processing and extraction of concept hierarchies.
- Once the text is extracted from the txt file, it is processed by applying various analysis techniques such as tokenization (breaking down text into individual words or phrases), sentence chunking (dividing into chunks or groups of words forming a sentence), part-of-speech tagging (identifying the grammatical role of each word), recognizing Roman literals (to recognize elements inside the table of contents), and identifying named entities using information from DBpedia.
- In the next step, the components such as the Table of Contents (TOC) including Chapter, Part, Subchapter, Subsubchapter, and other specific elements such as regulation name (REG), DBpedia concept (DBp), relationships (REL), and references (REF) are marked using rule-based methods.
- Within the document, all references are aligned with each TOC component based on the section boundaries. Additionally, information related to the citation summary (CS) components [regulation name, DBpedia concept, relationship, and sentence containing the reference (=context)] is pulled from the annotated file and connected to the REF (reference).
- Once all feature information is detected, it is saved in a simplified, flat format. Each line consists of one REF instance along with its associated TOC and CS features.

Since the originally proposed extraction method with the tool GATE, the approach has evolved into a programmatic solution involving Apache UIMA Ruta, custom Java, and Python components (Wehnert et al., 2019a). Aside from the omission of DBpedia concepts nowadays, the core components remain equal.

#### 2.1.3 Problems in concept hierarchy visualizations

There are several issues in visualizing concept hierarchies in general, including the following:

**Complexity (Noy and McGuinness**, **2001****):** Concept hierarchies can become quite complex, with many levels and a large number of concepts. This can make it difficult to understand the relationships between concepts and how to navigate the hierarchy.

**Ambiguity (Nguyen and Huang**, **2005****):** Concepts can have multiple meanings, and relationships between concepts can be ambiguous. This can make it difficult to determine the correct relationships between concepts in a hierarchy.

**Overlapping concepts (Guarino**, **1998****):** Concepts can overlap and share similar properties, making it difficult to distinguish between them in a hierarchy.

**Multiple hierarchies (Noy and McGuinness**, **2001****):** A concept can belong to multiple hierarchies, making it difficult to determine the correct placement of a concept within a hierarchy.

**Scalability (Uschold and Gruninger**, **1996****):** Visualizing large-scale concept hierarchies can be a challenging task. As the number of concepts and relationships increases, it can become increasingly difficult to effectively display and understand the hierarchy.

**Limited visualization techniques (Uschold and Gruninger**, **1996****):** There is a limited number of techniques available for visualizing concept hierarchies, each with its own strengths and weaknesses. Choosing the right technique for a specific application can be difficult.

Acknowledging these listed challenges, we choose to focus in this study on issues related to the visualization only (i.e., complexity, scalability, and limited visualization techniques). The remaining challenges affect the knowledge engineering process in general and are related to the content of those hierarchies, thus out of scope for this study.

### 2.2 Related work

In our literature search, we found different hierarchical data visualization methods for concept hierarchies and general hierarchical data. We then tested common visualization techniques using textbook data.

#### 2.2.1 Concept hierarchy visualization

Visualizing concept hierarchies commonly employs TreeMaps, showcasing hierarchical structures with the broadest concepts at the top and the most specific at the bottom (Johnson and Shneiderman, 1991). These diagrams effectively represent various hierarchical relationships, including is-a and part-of connections.

Concept maps, employing labeled arrows to depict relationships between concepts, excel in illustrating complex connections and interrelations among concepts (Novak et al., 1984).

Mindmaps can also be used to visualize concept hierarchies, with a central idea branching out to related concepts, aid brainstorming, and idea organization (Buzan and Buzan, 2006).

Additionally, graphical notations such as Entity-Relationship diagrams and UML diagrams find wide application in database and software engineering (Chen, 1976).

#### 2.2.2 Hierarchical visualization

Shneiderman's taxonomy, foundational in visualizing hierarchical data (Shneiderman, 1996), outlines the “overview first, zoom and filter, then details-on-demand” approach, widely adopted in data visualization. It suggests user-controlled interactions and emphasizes customized data structures for effective implementation.

The Pygmy browser offers a minimal space-filling technique for hierarchies in constrained displays, ideal when users have specific targets in mind. Its intuitive design facilitates easy node navigation and traversal path tracking (Band and White, 2006), inspiring our visualization for efficient screen use.

Holten (2006)'s clutter-free hierarchical visualization combines TreeMaps and graph approaches, arranging data radially and bundling similar hierarchy paths. Despite limitations such as over-aggregation and computational intensity (Holten, 2006), it addresses large, complex datasets effectively.

Woodburn et al. (2019)'s study highlights ICICLE plots and sundown visualizations' superiority over TreeMaps in navigation and intuitiveness with quantitative data. Elmqvist and Fekete (2009) propose local aggregation methods for compact visualization, augmenting interactions for hierarchical exploration—useful for preliminary textbook selection pre-top-down traversal.

Combining techniques, Stasko and Zhang (2000)'s Focus + Context approach integrates radial layouts, space-filling methods, and fisheye distortion to offer detailed node inspection without losing overall hierarchy understanding. Breadcrumbs maintain users' spatial awareness by tracking their path.

Collectively, these studies underscore diverse aspects of hierarchical visualization, from optimizing screen space to efficiently managing large data volumes. Acknowledging the absence of a perfect solution, they offer insights into addressing various challenges.

#### 2.2.3 Common hierarchy visualization methods

In this section, we examine the positives and negatives of common hierarchy visualization approaches.

##### 2.2.3.1 Word trees

We built a visualization with part of the data we have using Word Trees^2^ by Google, which is a textual data visualizing tool for hierarchical data. This tool enables users to have a view of the overall hierarchy on the initial load while also providing drill-down and drill-up interaction features, along with zooming. The graphical visualization allows users to select a node at any level and displays subsequent levels based on the selection. It also shows the frequency of nodes present at each level.

**Advantages of word trees:**

**Figure 2:**
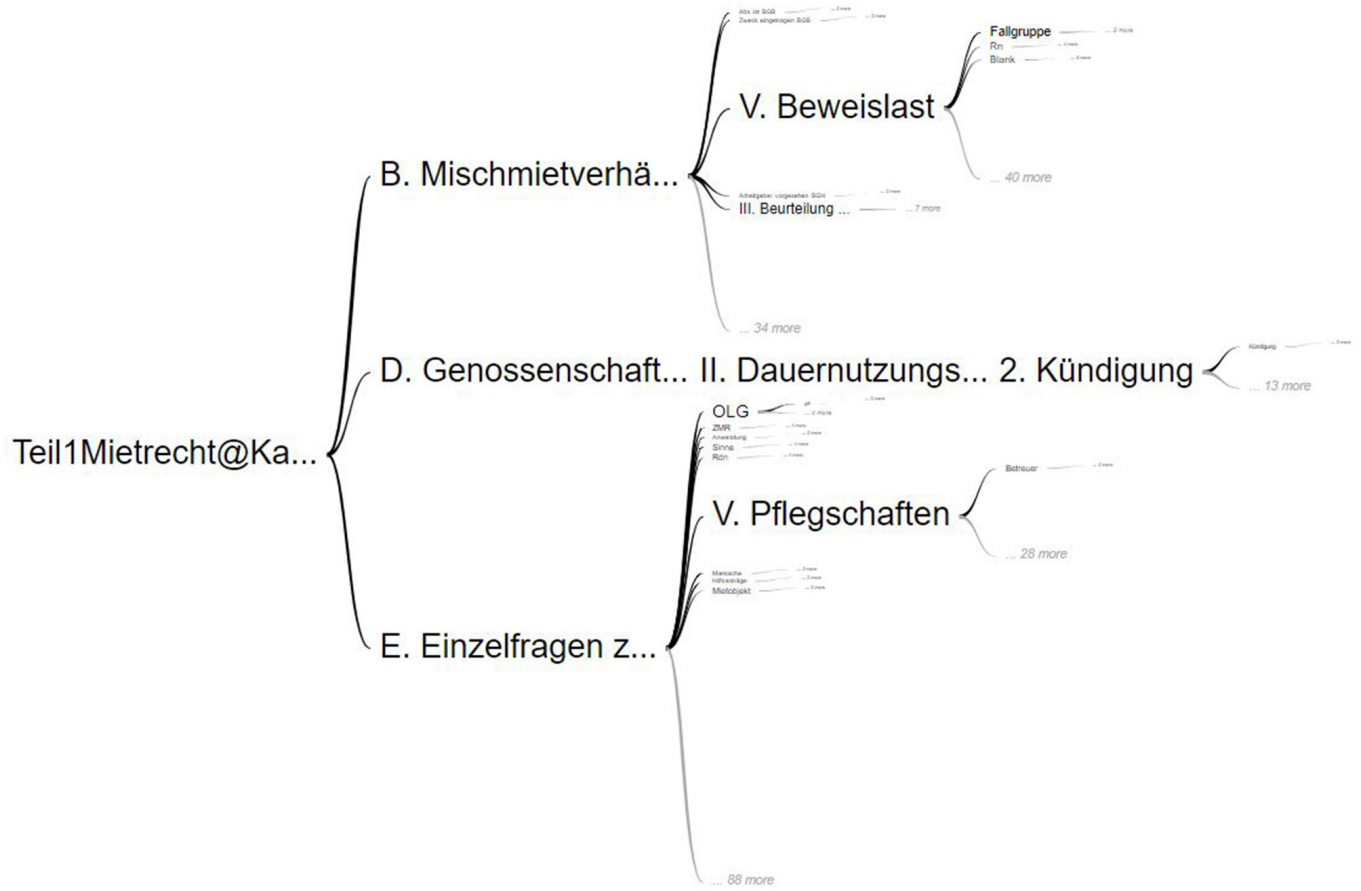
Google word tree with data from a legal textbook by Harz et al. (2018).

- **Easy visualization:** We found out that the visualization, as shown in Figure 2, is easy to interpret because of the representation of relationships that are shown along with the possibility to present the context (Wattenberg and Viégas, 2008).
- **Interactive:** It is possible to offer various interaction features such as drill-down and drill-up, while also showing the path traveled so far within the hierarchy and showing the subsequent levels. Users can also jump between different levels easily.
- **Flexible structure:** It provides a flexible structure that can accommodate hierarchies of varying levels. We were able to display a hierarchy with 12 levels without any issues.

**Disadvantages of word trees:**

**Figure 3:**
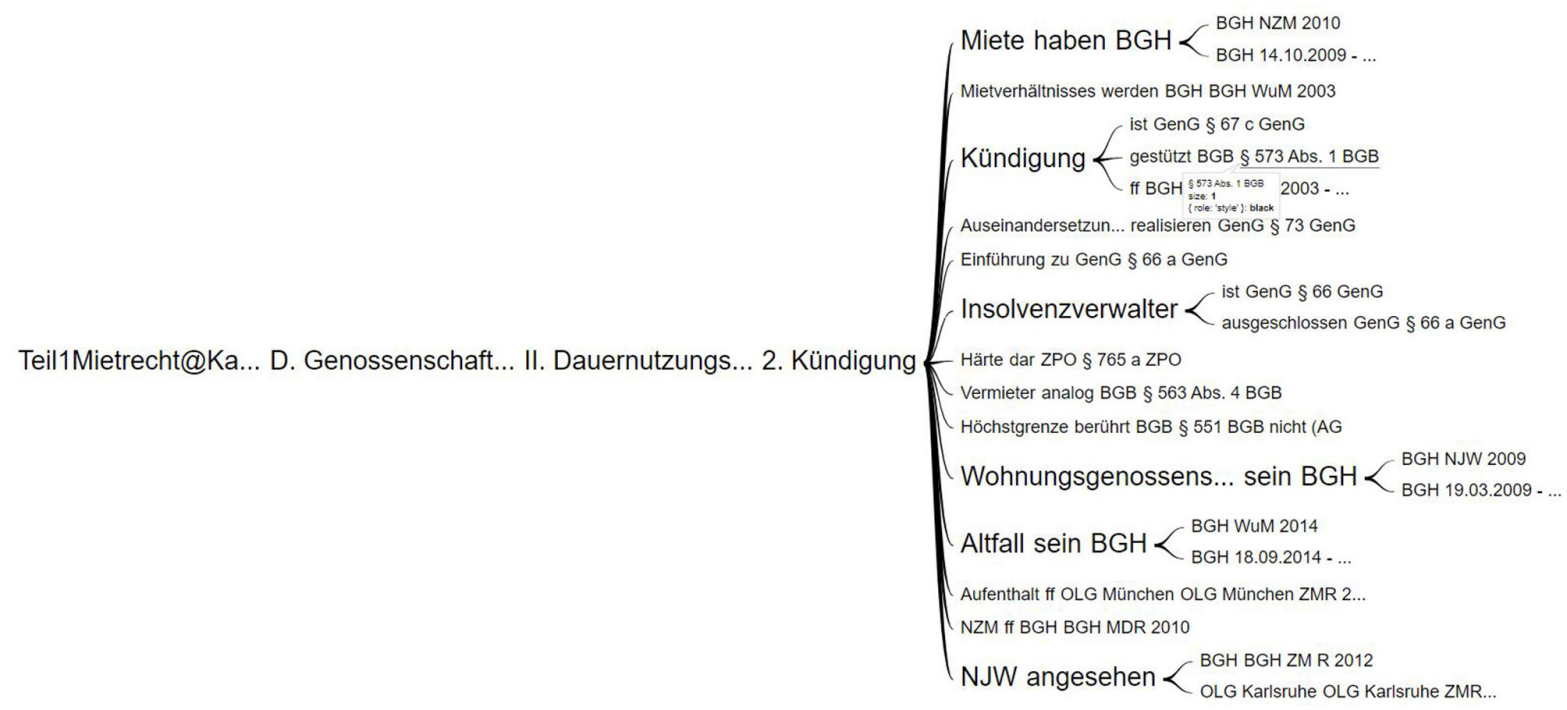
Google word tree in selection mode with data from a legal textbook by Harz et al. (2018).

- **Data overload:** When working with huge and complex datasets, the Word Tree gets cluttered and becomes difficult to read or interpret. This is noticed when interacting with hierarchies with more than 6 levels or when the nodes contain longer texts spanning multiple lines, which can be a required feature when working with hierarchies extracted from textbooks (e.g., to show the passage an entity was extracted from).
- **Over-simplification:** Ease of visualization is advantageous when working with simple hierarchies. For our use case, there are some functionalities in Word Trees that are not customizable, thereby limiting the implementation of complex features (e.g., filtering).
- **Missing the big picture:** Even though the entire hierarchies are displayed initially, as users traverse through the hierarchy, it stops showing the previous nodes and only shows the selected nodes. This is painful to the user when they want to explore the previous level. For this, users have to undo the selection of the previous node every time they need to explore it.
- **Responsive design:** The Word Tree visualization is a fixed layout design as shown in Figure 3. When working with data containing more levels, it involves a lot of scrolling and zooming out.

##### 2.2.3.2 TreeMaps

**Figure 4:**
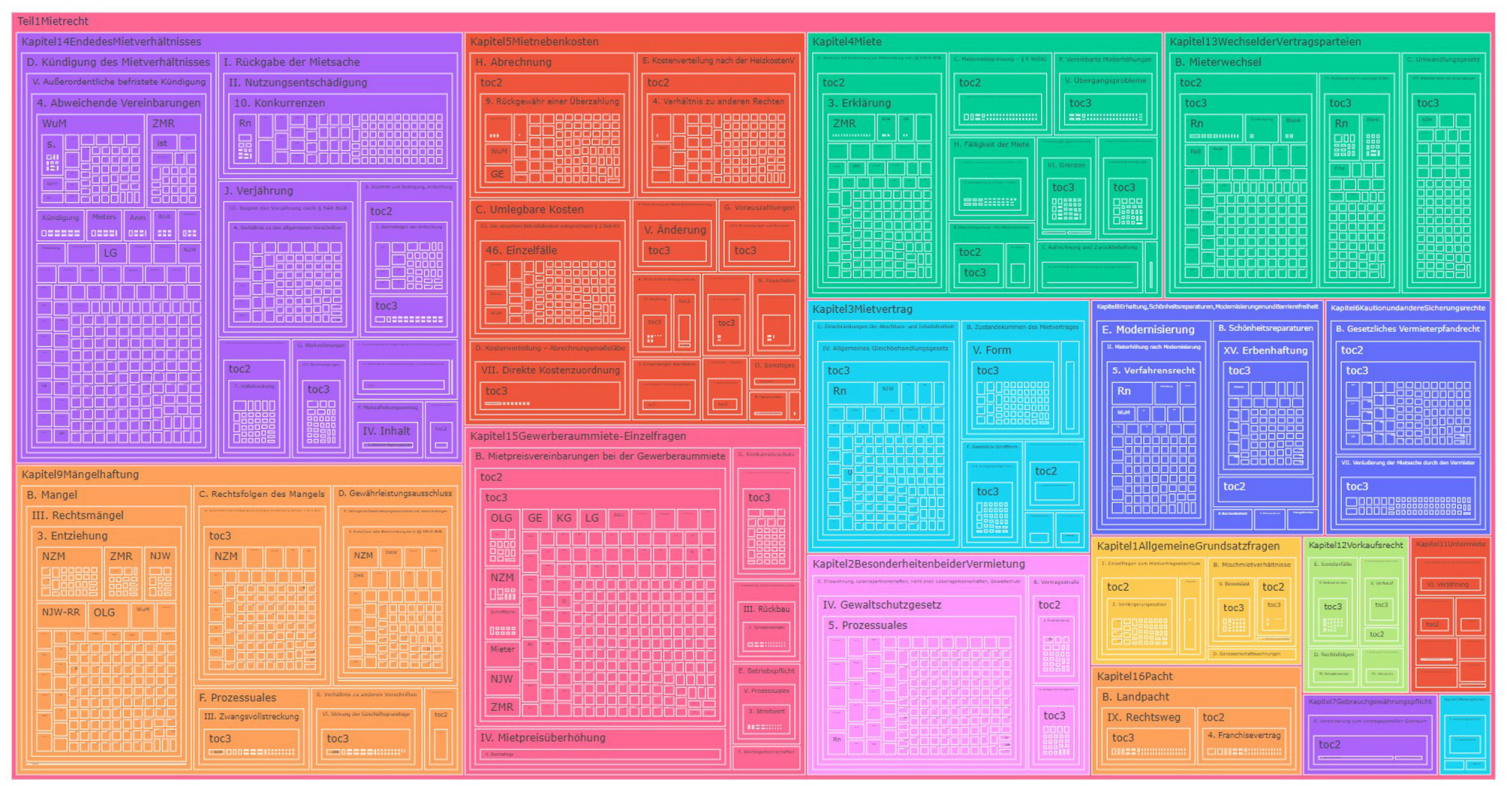
TreeMap with data from a legal textbook by Harz et al. (2018).

TreeMaps are considered the popular visualization when working with large volumes of data. In this visualization, the hierarchies are represented using rectangles, and the subsequent levels of hierarchy are represented using nested rectangles. The size of the rectangle corresponds to the size or number of levels it further contains. These are used when the data being visualized is considered to be too cluttered for traditional tree diagrams (Johnson and Shneiderman, 1991). We used a part of our dataset extracted from a textbook by Harz et al. (2018) to build Figure 4, and even with a small amount of data, the visualization becomes cluttered, difficult to read, and the lower hierarchical levels are barely recognizable.

**Advantages of TreeMaps**

- **Efficient use of space:** We observed that because of the way the hierarchies are organized as nested rectangles as shown in Figure 4, it conserves a lot of space, and a huge amount of data can be shown within the dimensions of the layout. The size of rectangles is dynamically adjusted based on the total available data and the size of the hierarchy.
- **Flexible structure:** TreeMaps in Figure 4 can be used to represent various datatypes in the same visualization. They are not rigid in the number of levels each hierarchy has and can accommodate varying levels dynamically.
- **Drill-up and drill-down interactions:** TreeMaps provide all the basic operations such as drill-down and drill-up interactions, along with the traveled path. They support filters and hover to view details.
- **Visual insights:** The possibility to use different colors for each hierarchy level makes it easier for a user to identify the levels and to establish connections between them easily.

**Disadvantages of TreeMaps**

- **Complexity:** Due to the way TreeMaps are visualized, as shown in Figure 4, it can be difficult to understand and interpret by users who do not have prior knowledge about this kind of visualization.
- **Unable to visualize the entire data:** Since the data are visualized in the form of nested rectangles, there is a limit on how many levels can be viewed at once.
- **Not suitable for textual data:** From our observation of a part of the dataset we have, a standard TreeMap is not suitable for deep hierarchies with our type of textual data, since the text spans are often longer, and it is not possible to show the entire text within the rectangle. In TreeMaps, we have to hover to see the full text.

##### 2.2.3.3 SunBurst

**Figure 5:**
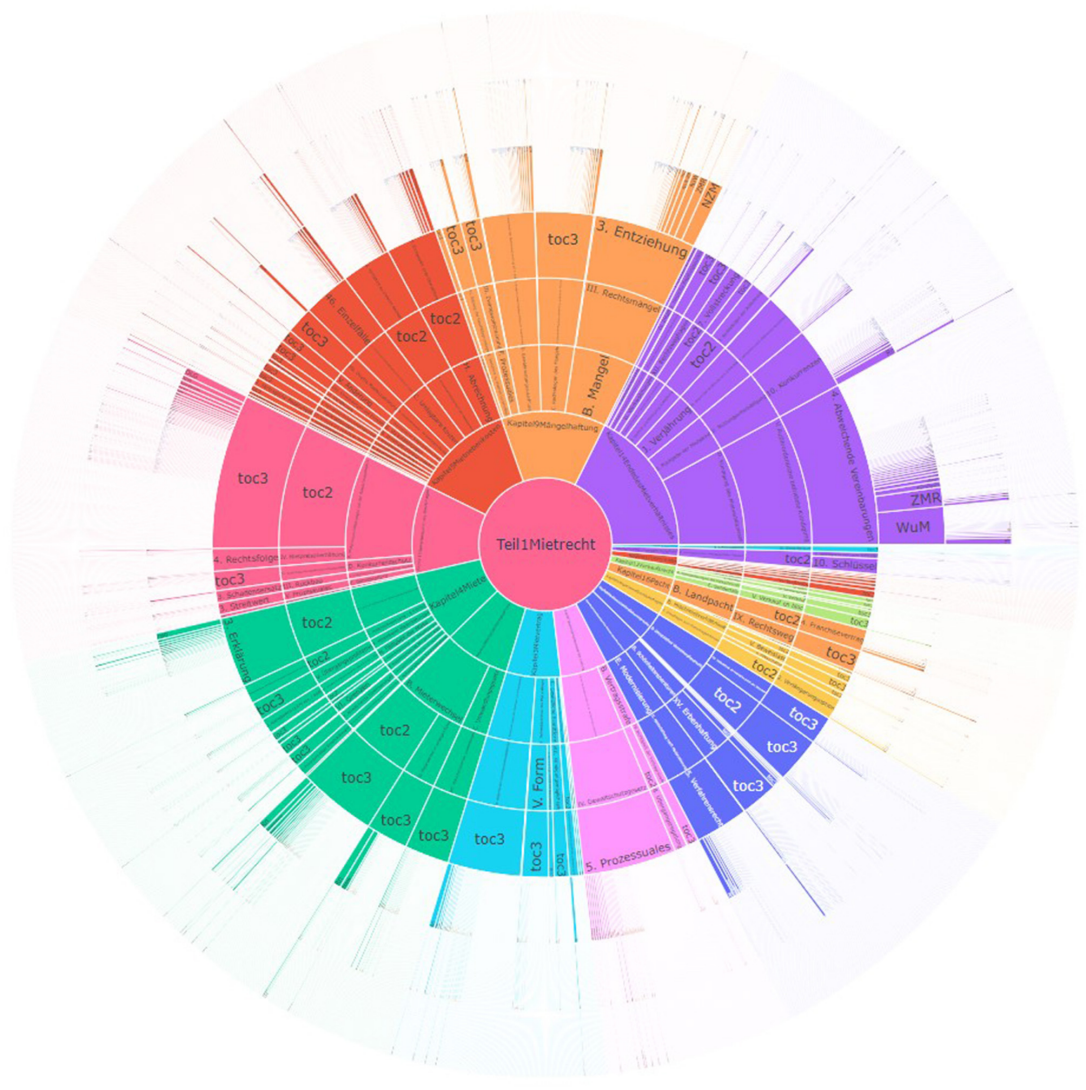
SunBurst diagram with data from a legal textbook by Harz et al. (2018).

SunBurst diagrams (Schroeder et al., 1998) are considered a radial space-filling visualization, as it incorporates rings in the visualization. The root node of the hierarchy is placed at the center, surrounded by the subsequent levels as concentric rings around the root node. In this way, one comes across more specific/detailed levels as one moves further away from the center. It provides drill-up and drill-down operations as its functionality and also supports top-down, bottom-up, and middle-out traversals. The selected node at any level will become the new center and the subsequent levels of that node are arranged as concentric rings around the selected node (Stasko and Zhang, 2000). We have built a SunBurst visualization in Figure 5 to better understand how it performs with a portion of our dataset (Harz et al., 2018).

**Advantages of SunBurst**

- **Compact visualizations:** Due to the way the SunBurst visualization presents the data, as shown in Figure 5, it is effective when representing large amounts of data. It can be used to get an overview of the volume of data.
- **Easy to compare hierarchies:** Due to the way the SunBurst visualization is organized, it is easier to compare hierarchies at multiple levels even without performing any operations.
- **Drill-up and drill-down interactions:** SunBurst offers fundamental functionalities such as drill-down and drill-up interactions, along with support for filter and hover actions to access detailed information.
- **Visual insights:** The possibility to use different colors for each hierarchy makes it easier for the user to identify the levels and to establish connections between them easily.

**Disadvantages of SunBurst**

- **Smaller outer levels:** When interacting with larger hierarchies with many levels, as shown in Figure 5, it becomes difficult to interpret the data as there is a limit on the number of levels that can be shown.
- **Readability issue:** When working with larger hierarchies or with textual data, due to the way the segment occupies space in the circle, it becomes impossible to read the text, as shown in Figure 5, unless the user hovers on the node. This becomes quite complicated when working with leaf nodes, as they do not get much space allocated.
- **Unable to see the path before the parent:** In the SunBurst visualization, the selected node is placed at the center and this causes an issue when traversing through the hierarchy, users can only go back to one level and cannot jump back to the root node or can see the path traveled so far.

##### 2.2.3.4 ICICLE plots

**Figure 6:**
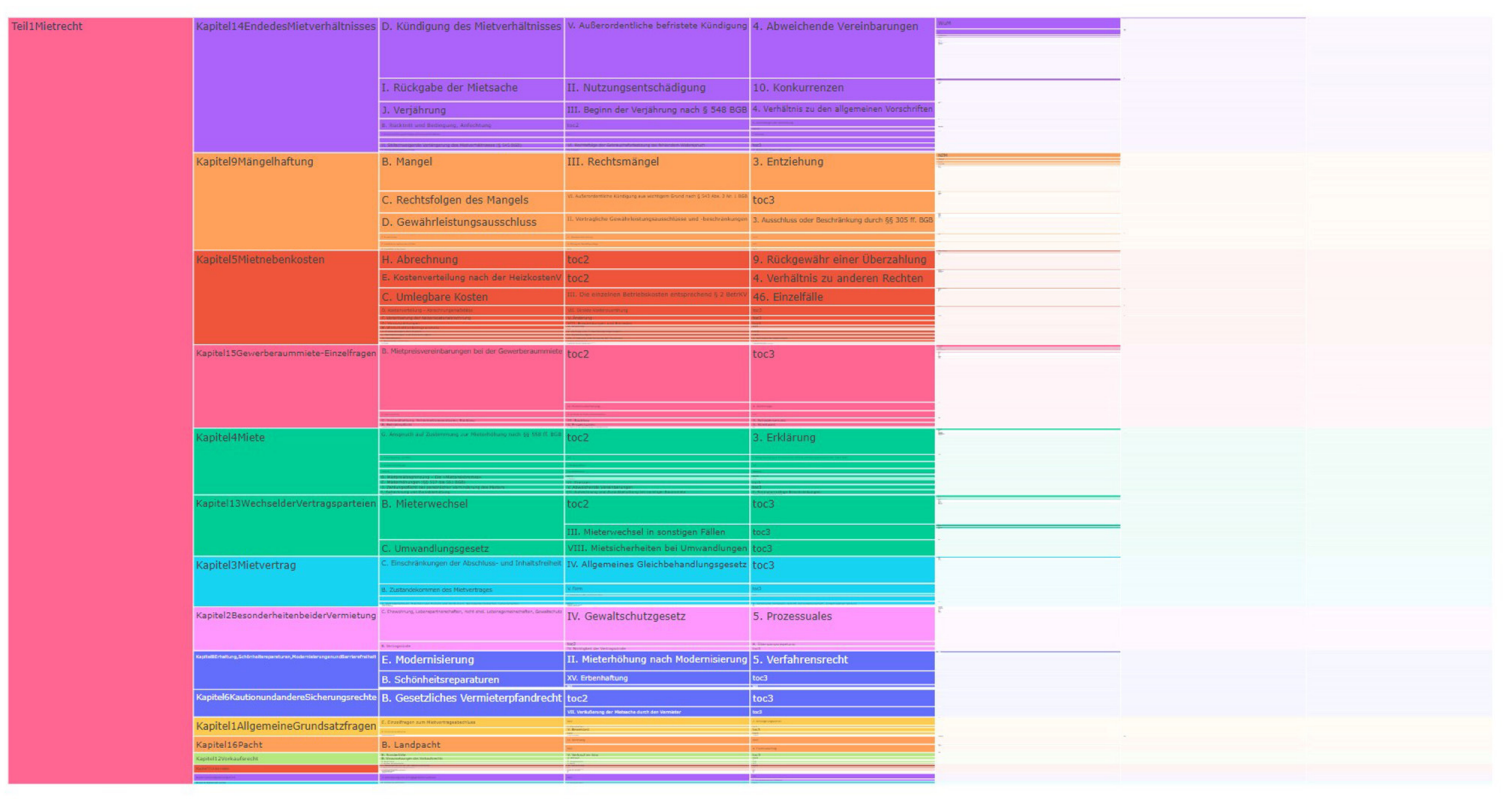
ICICLE plot with data from a legal textbook by Harz et al. (2018).

Another well-known visualization for hierarchical data are ICICLE plots (Kruskal and Landwehr, 1983). This visualization uses rectangles to show nodes and smaller rectangles that are stacked horizontally for subsequent levels. The length of each rectangle is determined by the number of nodes it has in the subsequent levels. In this visualization, the root node is displayed on the left and with subsequent levels placed toward the right. The hierarchies are placed from top to bottom. We have built an ICICLE visualization with a portion of our dataset (Harz et al., 2018) (see Figure 6).

**Advantages of ICICLE plot**

- **Efficient use of space:** ICICLE plots visualize the data as shown in Figure 6. They are considered very efficient when it comes to space utilization as they represent each node in the hierarchy using rectangles stacked side-by-side, which is also helpful when working with textual hierarchies.
- **Flexible structure:** ICICLE plots can be used to represent various data types in the same visualization. They are not rigid on the number of hierarchy levels and can accommodate varying levels dynamically.
- **Drill-up and drill-down interactions:** ICICLE provides basic operations such as drill-down and drill-up interactions, along with the traveled path. They also support filtering and hovers for details.
- **Visual insights:** There is the possibility to use different colors for each hierarchy and also levels.

**Disadvantages of ICICLE plots**

- **Smaller outer levels:** Navigating larger hierarchies with multiple levels, as depicted in Figure 6, poses challenges in interpreting the data due to limitations on the number of levels that can be displayed.
- **Readability issue:** Because of the way the rectangles take up space, reading the text becomes challenging unless the user hovers over the node. This complexity escalates when dealing with leaf nodes, which are allocated less space comparatively.

Note that the choice of visualization technique depends on the specific application and the level of detail required. To get an understanding of user needs, we follow the human-centered design process.

### 2.3 Methodology

#### 2.3.1 Human-centered design process

The human-centered design process (DIN EN ISO 9241-210, 2019) is a step-by-step approach employed by designers to understand user's needs to design and develop solutions that are in line with user preferences. This process has been adapted by application development to provide better applications as per the needs of users. The entire process is split into several iterative steps similar to other software development life cycles.

##### 2.3.1.1 Process

Following are the goals and tasks inside those steps.

**Empathize:** This step involves understanding the needs and experiences of the user by conducting research, observation, and other methods to identify their pain points. This step is crucial in creating designs that are focused on the user's needs and preferences (Pea, 1987).

**Define:** In this step, the data collected in the empathize stage is analyzed and the problem to be solved is defined. This step helps to ensure that the design process is focused and targeted toward a specific problem or issue (Holtzblatt and Beyer, 1997).

**Ideate:** This stage involves generating ideas and potential solutions to the defined problem. Brainstorming sessions, idea-generation methods, and other approaches may be used. This stage is about thinking creatively and generating as many potential solutions as possible (Brown, 2009).

**Prototype:** In this stage, physical or digital prototypes of the solutions are created to test and validate their potential effectiveness. This step allows designers to test their solutions in a low-risk, cost-efficient way, and make any necessary changes before moving on to the final stage (Gaver et al., 2003).

**Test:** In the final stage, the prototypes are tested with users to gather feedback and evaluate their effectiveness. This step is crucial in ensuring that the final design meets the needs and preferences of the user, such that any issues or problems are identified and addressed before the design is finalized (Rubin and Chisnell, 2008).

##### 2.3.1.2 Model-based approach in human-centered design

In human-centered design, a thorough grasp of user context is key. When information is lacking, further research via surveys or contextual interviews fills the gaps. Here, we adopt a model-based approach, as advocated by the “International Usability and User Experience Qualification Board” (UXQB e. V., 2023). This method allows us to work with incomplete user context data during the empathize phase, expediting prototype testing and feedback collection—aligned with Lean UX and Design Thinking principles (UXQB e. V., 2023). Our insight mainly stems from informal discussions with legal experts and students, offering a foundational understanding of their needs and challenges.

##### 2.3.1.3 Evaluation with usability studies

This section draws from Nielsen's work on usability (Nielsen, 1994), emphasizing its pivotal role in human-centered design. Usability studies enable designers to gather user feedback, observing interactions to pinpoint areas for enhancement. Techniques such as think-aloud protocols, surveys, and interviews, alongside metrics such as task completion rates and error rates, contribute to comprehensive usability assessments.

To ensure efficacy, diverse participant representation is crucial, paired with clear instructions and realistic tasks. Analyzing recorded user behavior uncovers patterns and issues, offering valuable insights to guide design decisions and enhance overall usability and effectiveness (Pruitt and Grudin, 2003). This process aligns with the principles of human-centered design.

#### 2.3.2 Empathize phase

This phase is very important for gaining information about how users feel while interacting with concept hierarchies and what kind of tool/visualization they need to explore these concept hierarchies easily. This phase includes observation, research, and user reviews to gain insights into the user needs, wants, and emotions (Brown and Wyatt, 2010). It is also important to observe users when interacting with the tool/visualization to understand their experiences when using it (Kouprie and Visser, 2009). For this, we have conducted extensive research and gathered information on our target users' needs and pain points both from online research and also from some users using the existing visualizations available for visualizing concept hierarchies.

##### 2.3.2.1 User needs

**Easy navigation within the hierarchy:** Users need an interface that allows them to easily navigate the hierarchy in various ways such as top-down, bottom-up, and middle-out traversal. This might include moving forward and backward between levels of the hierarchy, and moving laterally between nodes at the same level.

**Need for searchability:** When working with hierarchies that are text-based, users should have the possibility to search for specific concepts/text within the hierarchy. This should include the search for phrases or words in the nodes as well as in the context or references irrespective of their location in the hierarchy.

**Interactivity:** Enabling users to interact with the visualization should be straightforward and intuitive. This involves options such as filtering the hierarchy, accessing context as needed, and viewing all accessible leaf nodes from their current position. Additionally, it encompasses employing diverse interaction methods such as filtering, zooming, and on-demand detail display (Shneiderman, 1996).

**Scalability:** The visualization should perform the same even when the amount of data increases. This could mean that the interface should be able to handle large volumes of data, while still being clear and understandable. The visualization should not become cluttered when the number of levels in the hierarchy increases.

**Need to identify the levels easily:** Given that hierarchical visualization relies on multiple levels, it is essential for users to maintain their awareness of the current level and the path they have followed. This necessitates ensuring distinctiveness for each level within the hierarchy.

**Comprehension:** One such user need when working with the visualization of a concept hierarchy is that the visualization should help them identify the relationships that exist between the elements in the hierarchy. It is shown that the way in which hierarchy is presented to the user plays a major part in this. Related research (Stasko and Zhang, 2000; McGuffin and Jurisica, 2009) shows that some layout algorithms including TreeMaps and radial trees offer better comprehension compared to other hierarchical visualizations, such as node-graphs and ICICLE plots.

**Training and support:** Users should be provided with proper documentation that can help them use the visualization effectively.

To design a visualization that is suitable for exploring and visualizing concept hierarchies, we need to address all the user needs and also understand their pain points, presented in the following.

##### 2.3.2.2 Pain points

**Cognitive load:** Complex hierarchical visualizations can impose a high cognitive load on users, making it challenging for them to understand and process the information effectively. Studies have found that the choice of layout and the number of levels in the hierarchy can significantly impact users' cognitive load (Ghoniem et al., 2004).

**Visual clutter:** Visual clutter can result from overlapping elements, excessive use of colors, or dense visual representations, which can make it difficult for users to discern hierarchical relationships and identify important data points (Ellis and Dix, 2007).

**Navigation difficulties:** Navigating through complex hierarchical structures can be challenging, especially when users need to maintain a mental map of their position within the hierarchy. Research has shown that certain interaction techniques, such as zooming and panning, can help alleviate navigation difficulties, but these methods may not be sufficient for all users (Lam et al., 2017).

**Lack of customization options:** Users may have diverse preferences and requirements depending on their tasks and contexts. If a hierarchical visualization does not provide adequate customization options, it may fail to meet the specific needs of different users, leading to frustration and reduced effectiveness (Tory and Moller, 2004).

In summary, user pain points in hierarchical visualizations stem from factors such as cognitive load, visual clutter, navigation difficulties, and lack of customization options.

#### 2.3.3 Define phase

Following the human-centered design process, we define a problem statement that addresses the user needs and pain points we described in the empathize phase. This statement sets the direction in our research and development of the visualization.

##### 2.3.3.1 Problem statement

A dynamic, interactive system that allows users to explore and visualize concept hierarchies in top-down, bottom-up, and middle-out traversals efficiently and effectively is required. While reducing cognitive overload and visual clutter, the visualization should engage the users, while being easy to navigate and should be capable of scaling dynamically when new data are introduced.

##### 2.3.3.2 User persona

Following the problem statement, we have come up with various personas of our target users. In this study, we only present the persona type of law students which corresponds to the user group we invited for the usability test.

**Name:** Emily Fischer

**Age:** 25

**Occupation:** Law student

**Education:** Emily is currently pursuing a law degree.

**Location:** Düsseldorf

**Goals:**

- Understand complex legal concepts quickly and easily.
- Improve her ability to identify relationships between legal concepts.
- Increase her efficiency in studying legal textbooks.

**Frustrations:**

- Struggles to comprehend complex legal concepts.
- Difficulty identifying relationships between legal concepts.
- Finds it challenging to stay focused when reading dense legal textbooks.

**Motivations:**

- Wants to excel in her legal studies and future legal career.
- Enjoys finding new and innovative ways to approach legal research and study.

**Personality:**

- Analytical and detail-oriented.
- Open-minded and curious.
- Ambitious and driven.

**Behaviors:**

- Emily often spends long hours studying and researching legal concepts.
- She enjoys using technology to aid her in her studies.
- Emily values efficiency and is always looking for ways to improve her study process.

**Goals in relation to the interactive system:**

- Emily wants to use the interactive system to quickly understand complex legal concepts.
- She wants to use the interactive system to identify relationships between legal concepts more easily.
- Emily hopes that the interactive system will increase her efficiency when studying legal textbooks.

##### 2.3.3.3 User scenario

Creating user scenarios can help illustrate how different users might interact with the system for exploring and visualizing concept hierarchies in textbooks. Such a scenario describes the context, actions, and outcomes of user interaction.

**Scenario:** Emily, the Undergraduate Student

**Context:** Emily is studying for her upcoming law exam. She is struggling to understand the connections between various legal concepts and how they fit into the overall topic.

**Action:** Emily opens her digital textbook and navigates to the dynamic concept hierarchy visualization. She starts with a high-level view, clicking on a main concept to reveal its sub-concepts. She uses interactive features to explore the connections between concepts, viewing references and context as she goes.

**Outcome:** Emily gets a better understanding of the topic's structure and feels more prepared for her exam. The visualization makes it easier for her to remember and understand the connections between different concepts. She can view the norms and legal cases associated with a topic and its sub-topics, based on where they were cited in the textbook that she explores.

#### 2.3.4 Ideate phase

The Ideate phase can be considered a critical juncture where all the insights gathered from the understanding of the user needs and pain points are combined to propose possible solutions. These solutions are presented as concepts or ideas that are considered foundations for prototyping and testing.

We quickly realized that using traditional methods such as Word Trees, TreeMaps, SunBursts, and ICICLE plots will not be helpful in providing insights into the structure and content of our hierarchy, which is why we now define user requirements for each traversal direction to develop a custom prototype.

##### 2.3.4.1 Top-down traversal

- **REQ1-Book and chapter overview:** Users should be able to select a specific textbook and get an overview of its chapters.
- **REQ2-See references (leaf nodes) at all times:** On selecting the chapters, users should be able to see all references that belong to the chapter or any other textbook section deeper down in the hierarchy.
- **REQ3-See the context of references on demand:** Users should be able to see the context in which the particular reference is cited.
- **REQ4-Drill up, drill down, and filter:** Users should be able to perform all the basic navigation techniques drill up, drill down, and filter options at any point inside the hierarchy.
- **REQ5-Have an overview of the traveled path:** At any level of the hierarchy, users should have the ability to look at the path traveled so far, along with an option to explore and jump to any previous level in the selection.
- **REQ6-Visual insights:** Users should be able to identify the level inside the hierarchy just by looking at it. Hence, there is a need to distinguish each level across the visualization which will help them as a visual reminder of the level they are in.
- **REQ7-Dynamically scalable:** The user should be able to incorporate hierarchies without any issues into the visualization, irrespective of the number of levels they have.

##### 2.3.4.2 Middle-out traversal

- **REQ8-Search in context or node:** Since the idea of the middle-out traversal approach is to be able to start from anywhere within the hierarchy, users should be able to search for a node or term within the hierarchy to begin, which shall give them an overview of the results of that searched term occurring in context or in the hierarchy nodes.
- **REQ9-Show the path from the root to a selected node:** It is important to see the hierarchy path to the searched node, which will keep users informed about the hierarchy level they are in. This also helps users in understanding the level at which this node occurs and how it relates to other concepts of the same or different levels.
- **REQ10-Reference and context:** Displaying references along with the context can help users in understanding the link between the concepts and the reference.
- **REQ11-Ability to explore the siblings at each level:** When interacting with a concept, a user should also be able to explore all the sibling nodes at each level.
- **REQ12-Finding similar leaf nodes:** Users should be able to see other nodes with the same reference.
- **REQ13-Drill up, drill down, and filter:** Users should be able to perform all the basic navigation techniques such as drill up, drill down, and filter options at any point in the hierarchy.
- **REQ14-Visual insights:** Users should be able to identify the level of hierarchy just by looking at it. Hence, there is a need to distinguish each level across the visualization which will help them as a visual reminder of the level they are in.
- **REQ15-Dynamically scalable:** The visualization should support hierarchies with varying levels without any difficulty. When users search for a keyword, it should also search for that keyword in all the hierarchies (e.g., When a new hierarchy having two more levels than the existing levels is introduced, the visualization should be able to extend to show the new levels, as well).

##### 2.3.4.3 Bottom-up traversal

- **REQ16-Search and filtering:** In bottom-up traversal, users begin at leaf nodes, and usually, the number of leaf nodes in the hierarchy is large. Hence, users should be able to search for the nodes they are looking for and to filter the nodes.
- **REQ17-Visual insights:** The visualization should allow users to identify the level in the hierarchy easily, and help users in understanding how the reference is used at various levels, in different contexts.
- **REQ18-Drill up, drill down, and filter:** When users start their search with references, they should be able to move up in the hierarchy to find the relevant parent nodes. To facilitate this, there should be a possibility to perform all the basic navigation techniques such as drill up, drill down, and filter options at any point in the hierarchy.
- **REQ19-Overview of occurrence:** Users should be able to see all the occurrences of the particular leaf node in all the available hierarchies.
- **REQ20-Path highlighting:** Users should be able to see the hierarchy path for the selected node and should be able to jump to any level in the path.

#### 2.3.5 Prototype phase

In this section, we present the design to address each requirement for our use case.

##### 2.3.5.1 Requirement-based design

###### 2.3.5.1.1 Top-down traversal

**Figure 7:**
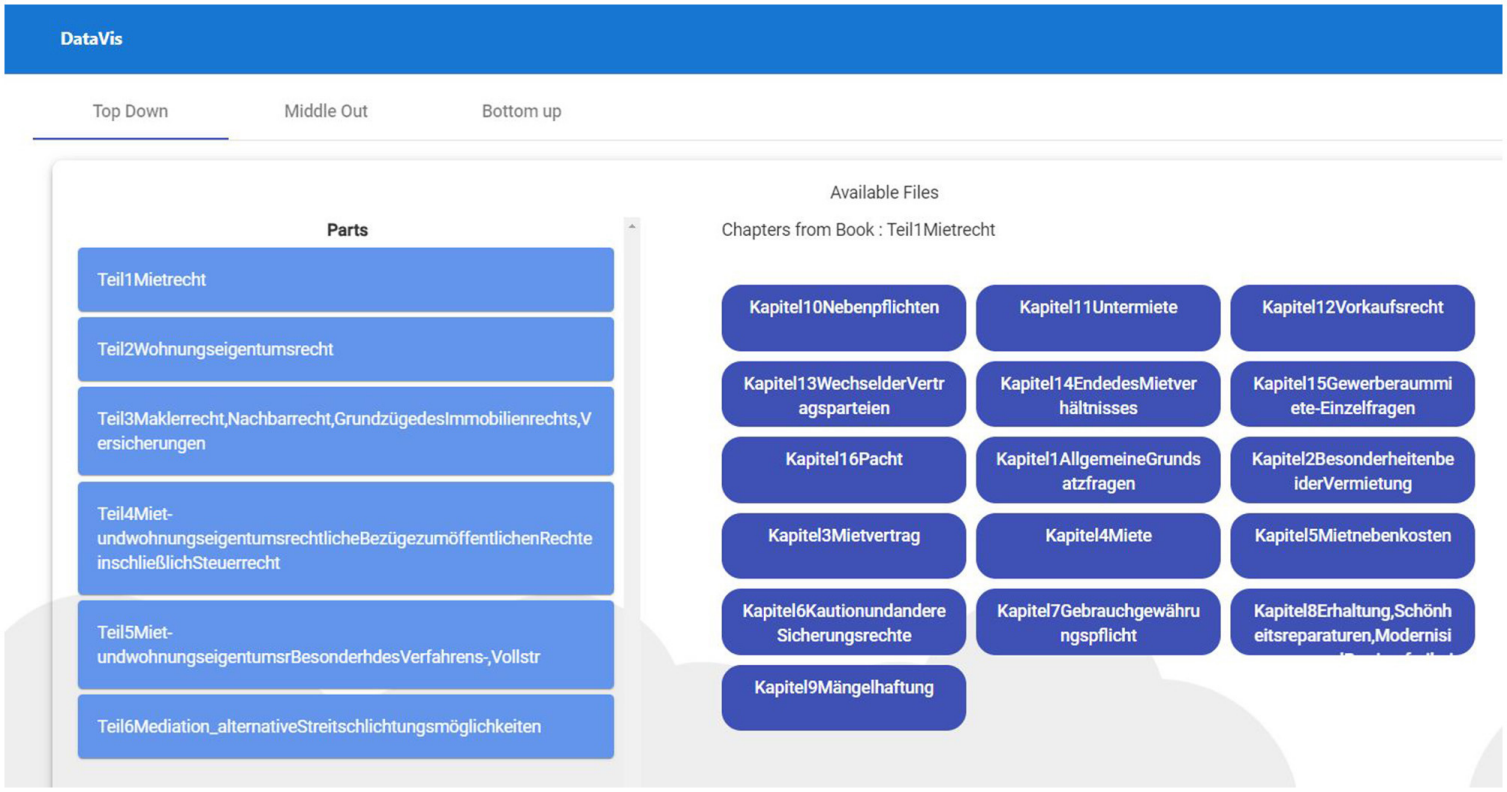
Top-down visualization with data from a legal textbook by Harz et al. (2018).

**REQ1-Book and chapter overview:** To address this requirement, we have developed a visualization that will also be starting point of the visualization for top-down traversal. In this visualization, we display all the books that are available on the left side of the screen, as shown in Figure 7, and once the user clicks on any of the displayed books, we will then load all the chapters that belong to the selected book.

**Figure 8:**
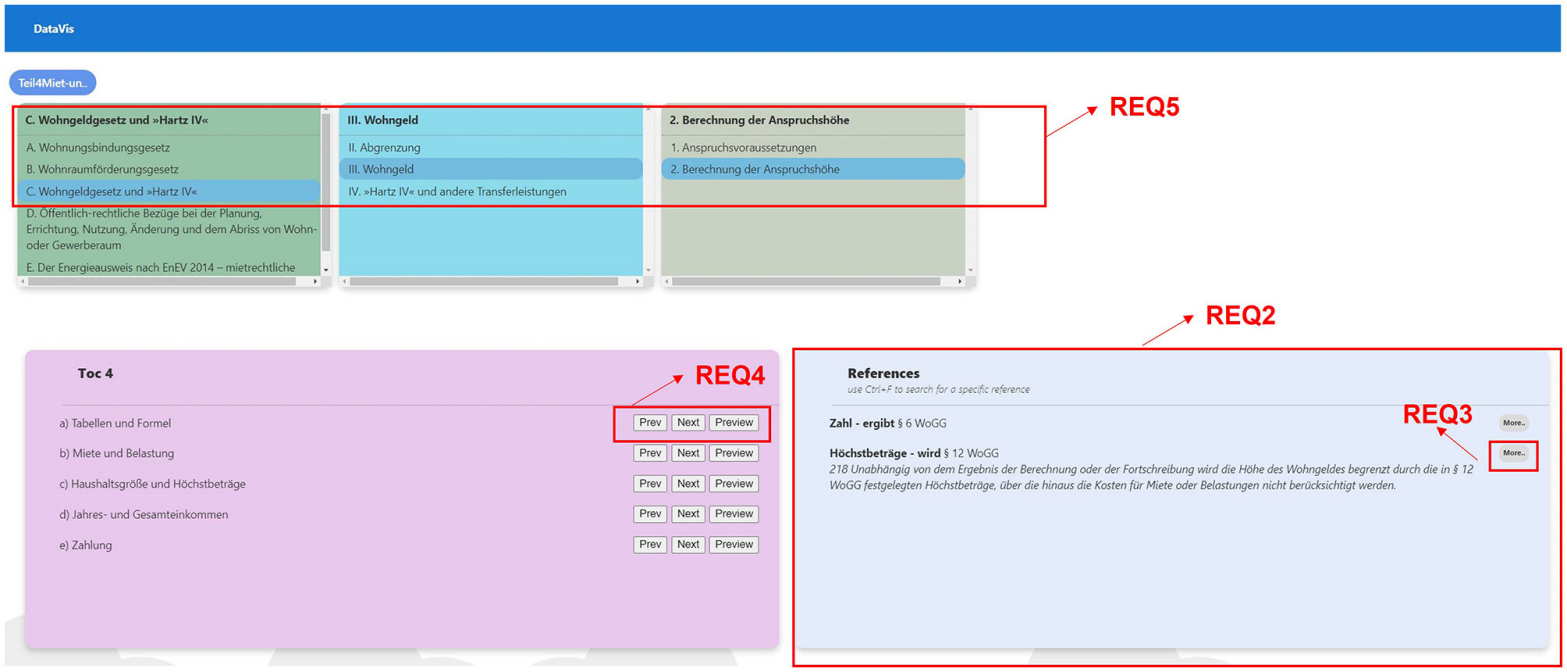
TOC view requirements with data from a legal textbook by Harz et al. (2018).

**REQ2-See references (leaf nodes) at all times:** To address REQ2, we have designed a dedicated area in the “TOC” view to show the references based on the selected concept/chapter at all times. Figure 8 depicts the implementation of REQ2.

**REQ3-See the context of references on demand:** For showing the context in which the references are cited, we came up with the idea of having a “More” button that reveals the context when clicked (see Figure 8), REQ3.

**REQ4-Drill up, drill down, and filter:** To satisfy REQ4, we have designed a button group with three buttons “Next (for drill-down), Prev (for drill-up), and Preview (for filtering references)” that are displayed beside each text from within the TOC at the current level, as illustrated by REQ4 of Figure 8.

**REQ5-Have an overview of the traveled path:** To fulfill REQ5, we have come up with a design that shows the traversed TOC on the top of each previous level in the “TOC” view, along with highlighting the selected TOC, as shown in REQ5 of Figure 8.

**REQ6-Visual insights:** To help users identify the level in the hierarchy easily, we have come up with a design that assigns a unique color for each TOC level across the application, as shown in Figure 8.

**REQ7-Dynamically scalable:** To address REQ7, we have implemented the logic in such a way that there is a periodic scan for new books or hierarchies available in the database to processes the data accordingly.

###### 2.3.5.1.2 Middle-out traversal

**Figure 9:**
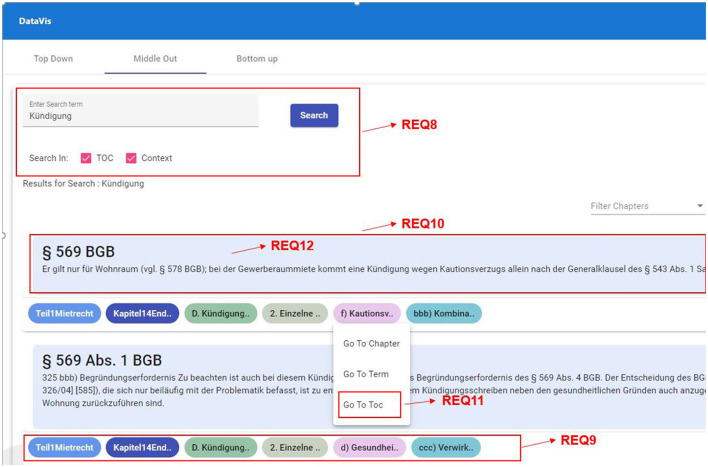
Middle-out traversal requirements with data from a legal textbook by Harz et al. (2018).

**REQ8-Search in context or node:** As depicted in Figure 9, we have designed a search box and a checklist box to enable the user to select where the search term should appear in the hierarchy. Once the user clicks on the search button, the results are loaded based on their search criteria.

**REQ9-Show the path from root to a selected node:** To enable the user to quickly understand where the searched term is appearing in different hierarchies, we show the path from root to the selected node using small chips-like icons below each result as shown in Figure 9, REQ9.

**REQ10-Reference and context:** We show all the references assosciated with the searched keyword, by displaying a list of references and the context in which the reference is used, as shown in Figure 9, REQ10.

**Figure 10:**
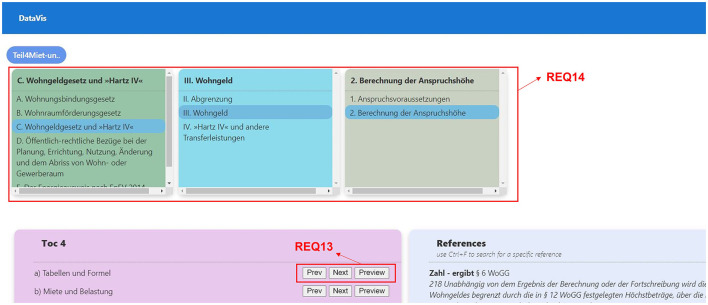
Middle-out traversal requirements, TOC mode, data from a legal textbook by Harz et al. (2018).

**REQ11-Ability to explore the siblings at each level:** As shown in Figure 9, we have designed the visualization to allow users to explore siblings of any selected node by clicking on the node that will display a menu of options, clicking on the menu item “go to TOC” will take users to the TOC view to explore the sibling nodes, as shown in Figure 10.

**REQ12-Finding similar leaf nodes:** We have integrated an option for users to search for similar references that are used in various contexts by clicking on the reference (see Figure 9) that will load all the results containing the same reference as a leaf node in various hierarchies.

**REQ13-Drill up, drill down, and filter:** As shown in Figure 10, we have provided an option for users to further explore any hierarchy by either using “Prev (for drill up), Next (for drill down), and Preview (for filtering).”

**REQ14-Visual insights:** To enable users to identify the hierarchy level easily across the application, we have assigned a unique color to represent a hierarchical level (see Figure 10).

**REQ15-Dynamically scalable:** We have designed the visualization to scale dynamically, by checking for the number of levels available for each search result and to display the elements accordingly.

###### 2.3.5.1.3 Bottom-up traversal

**Figure 11:**
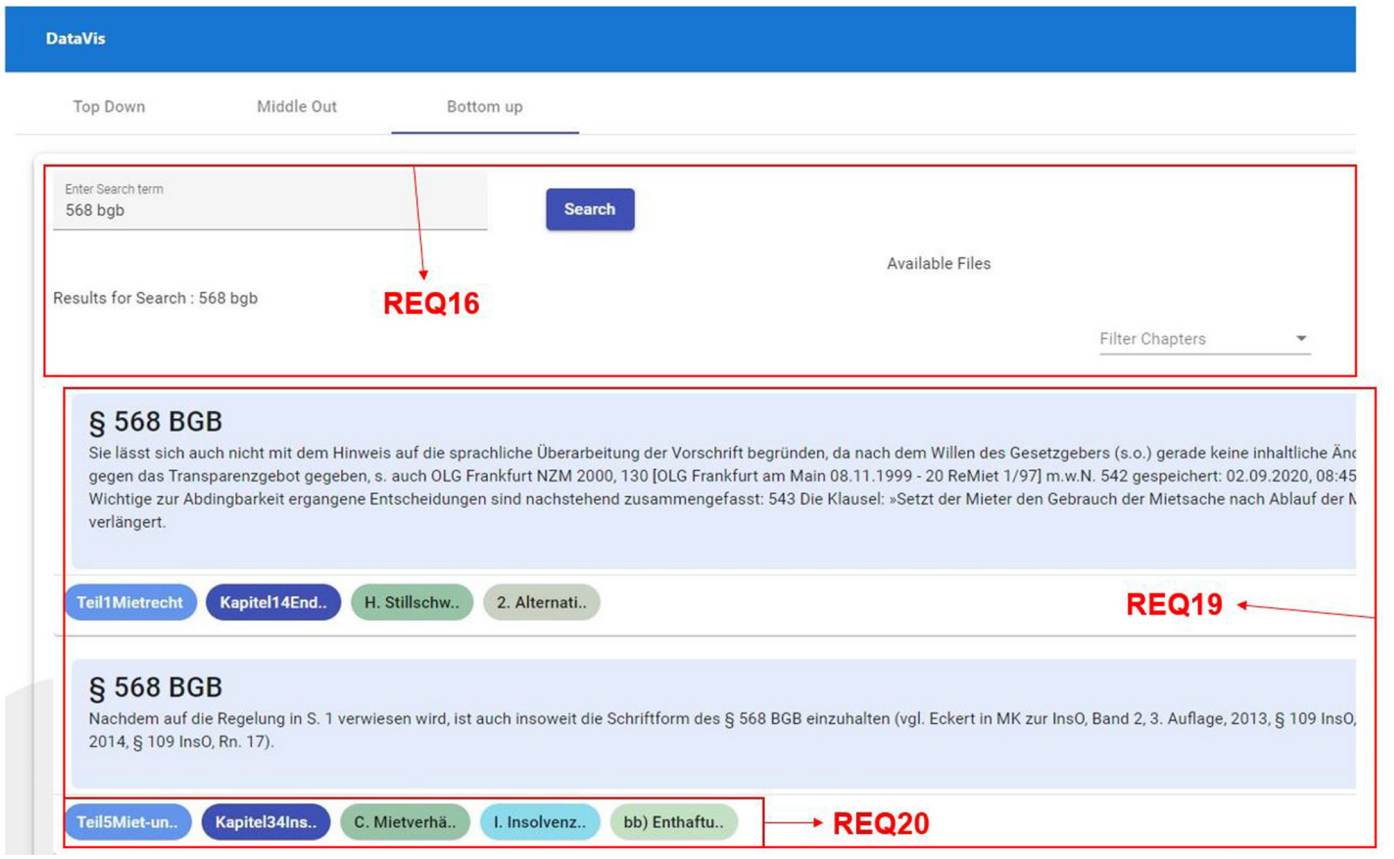
Bottom-up traversal requirements with data from a legal textbook by Harz et al. (2018).

**REQ16-Search and filtering:** To facilitate the search for a specific leaf node, we enable full and partial text matches and provide a filter functionality that lets the users filter by book or part name as shown in Figure 11, REQ16.

**Figure 12:**
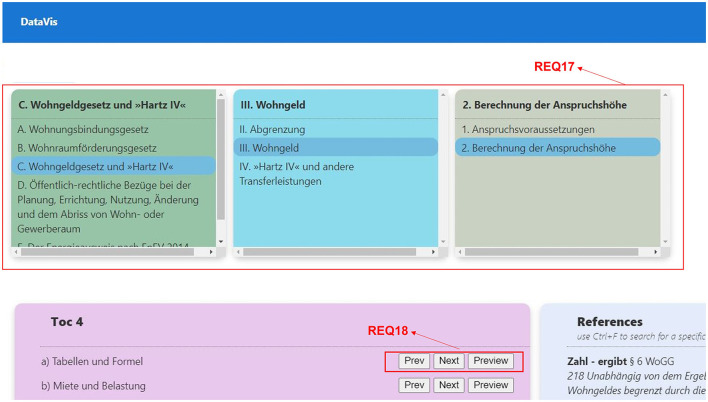
Bottom-up traversal requirements, TOC mode, data from a legal textbook by Harz et al. (2018).

**REQ17-Visual insights:** This is one of the common requirements across all three traversal techniques. We have designed the system to use a unique color to represent each hierarchy level, thereby making it easier for users to identify the level across the visualization, as shown in Figure 12, REQ17.

**REQ18-Drill up, drill down:** This is another common requirement across all three traversal techniques. We have designed a button group that is displayed beside each TOC, which will help users in either moving up or moving down the hierarchy along with a preview option that filters the references of a selected hierarchy, as shown in Figure 12, REQ18.

**REQ19-Overview of occurences:** We designed the interface to show all the occurrences of searched references as an overview for the user, for understanding the various contexts in which the particular reference is used, as shown in Figure 11, REQ19.

**REQ20-Path highlighting:** We show the path from the root to the node in which the reference occurs at the bottom of each search result to inform users how and where the reference is used within the hierarchy, as depicted in Figure 11, REQ20.

In this way, we were able to design our visualization that addresses all the requirements defined during the ideate phase, given our research questions.

##### 2.3.5.2 Implementation

For the front end of this visualization, we used platform-independent angular 10^3^ along with Material UI^4^ components. In this framework, there is a clear separation between the front-end and back-end parts. We have chosen Orient DB^5^ as our database, as it supports the use of multi-model databases such as document type, key-value type, and object models along with supporting SQL queries. Another reason to choose Orient DB is that the data can be requested through a REST call, which provides the flexibility of choosing a programming language that suits our needs rather than restricting ourselves to the languages that support Orient DB.

###### 2.3.5.2.1 Research question 1

**“Which visualization is suitable for a top-down search for hierarchical data?”**

To answer our first research question (which is about finding a suitable visualization that supports Top-Down traversal), we explored various visualizations and understood that the current visualizations work only for some hierarchical data formats. Hence, we have come up with a visualization approach to address the user needs and pain points we have gathered. We created a high-fidelity prototype of the visualization we have designed in the ideate phase. This prototype is explained next.

**Book and chapter overview:** In Figure 7, the panel on the left-hand side displays books that are available for visualization. The blue rectangles on the right display the chapters of the selected book. This is the starting point of the top-down visualization where the user starts with the book he or she likes to explore further. When users click on the chapter, they are presented with all the available hierarchies that belong to the selected chapter.

**See references (leaf nodes) at all times:** We show all available references on the right at all times based on the selected level. The current level is always displayed as a big box on the left.

**Drill up, drill down, and filter:** Three buttons that are present beside each node provide options to drill down, drill up, and filter.

- “Next” is used to navigate to the next lower level (drill down) of the selected hierarchy.
- “Prev” is used to navigate to the parent node or previous higher level of the selected hierarchy.
- “Preview” is used to filter the references that belong to the selected node in the hierarchy.

**Have an overview of traveled path:** Users are presented with an overview of the selected nodes in smaller boxes on the top when using the drill-down operation.

**Visual insights:** Each level of the hierarchy is represented using a unique color to help users identify the levels easily.

**See the context of references on demand:** The “More” option available beside each reference can be used to see the context in which the reference is used (i.e., the sentence which contains the reference).

###### 2.3.5.2.2 Research question 2

**“Which visualization is suitable for a middle-out search for hierarchical data?”**

To approach our second research question, we introduced a blended visualization strategy, addressing the identified user needs and pain points. A prototype representing our envisioned visualization was developed during our ideation phase. This will be further discussed.

**Search in context or node:** Using the middle-out search bar, users will be able to search for the chapter or term they are interested in. They also will have the possibility to select if the results should contain the search term either in the reference context or in the TOC (table of contents) element, or both. Results are visualized in such a way that they contain the following elements:

- Relationship to the reference.
- Reference.
- Context.
- Path from the name of the book to the node where the reference occurs.

**Show path from the root to the selected node:** See research question 1.

**Reference and context:** In the middle-out visualization, we provide the user with the ability to see all the leaf nodes from all the hierarchies that contain the selected term, either as part of nodes in the hierarchy or in the context. This includes the reference and the context in which the reference is used.

**Ability to explore the siblings at each level:** From our research, we observed that users need the possibility to also explore all the sibling nodes at each level. To accommodate that, we provide users with an option to select any node in the hierarchy path shown in the result. This will take them to the drill-down view where users can explore siblings of the selected node along with all references at the selected level. This view also provides users with the possibility of exploring siblings at all the previous levels.

**Finding similar leaf nodes:** Users can click on the reference shown in the result which will show all the occurrences of that reference in other hierarchies. This will help users in identifying the relationship to other topics.

**Drill up, drill down, and filter:** This is one of the common functionalities across all three traversal techniques, and in order to accommodate these options in the middle-out visualization, we have introduced these three options beside each TOC element at the current level, as shown in Figure 9. Using these buttons, users can navigate within the hierarchy in both directions, as well as filter the references.

**Visual insights:** Each level of the hierarchy is represented using a unique color to help users identify the levels easily, see also research question 1.

**Dynamically scalable:** This visualization is designed with scalability in mind, such that it can incorporate all the new hierarchies coming in and works the same. It can also accommodate new levels in the hierarchy with ease.

The HTML part of the initial view contains an input form where users can enter the concept/term they would like to known more about. It also provides users the flexibility of stating where the term should appear in the search results (as part of TOC or as part of the context). On submitting the inputs, the users are then presented with a list of results as shown in Figure 9, where each result is divided into three parts:

- Reference and context in the center.
- The path from the root (book) to the specific level in which this result appears in the bottom.

This path is shown as chips at the bottom of each result. These chips are clickable and reveal various options based on the chip, such as go to chapter, go to TOC, and search for the term, as shown in Figure 9.

###### 2.3.5.2.3 Research question 3

**“Which visualization is suitable for a bottom-up search for hierarchical data?”**

To address our third research question, which seeks an apt visualization facilitating a bottom-up approach, we examined multiple visualization techniques. We found that existing visualizations are suitable for certain hierarchical structures, but frequently lack in answering the challenges and needs users encounter with text-based hierarchical data. Therefore, we proposed a combined visualization technique to meet the challenges and needs identified.

**Search and filtering:** Users will be presented with all the leaf nodes (references) based on their input. This will display all the results that contain the full term or part of the search term from all the hierarchies. Alternatively, users can also look for all occurrences of the particular leaf node by clicking on the reference in the middle-out approach. In this way, users can see all the different contexts in which the reference has been used.

**Visual insights:** See research question 2.

**Drill up, drill down:** Users can move up (drill up) and move down (drill down) the hierarchy levels by clicking on the “Next” and “Prev” buttons placed beside each TOC. In addition to this, users can also filter all the references that belong to a TOC by clicking on “Preview” button that is grouped along with “Next” and “Prev” buttons.

**Path highlighting:** The results shown will contain the path from the book name (root node) to the node where the reference occurs. This path is shown at the bottom of each reference using chips-like structures, see also research question 2.

This view is similar to middle-out view; however, there are slight differences in the functionality as the text entered in the search field will only search for the term in references of the hierarchy and lists results, where each result is divided into two parts.

- Reference and context in the center.
- Path from the root (book) to the specific level in which this result appears in the bottom.

To sum it up, we have created a common TOC view for all three visualizations and implemented three different starting points for different traversal approaches.

## 3 Results

We evaluated the application's effectiveness using three interconnected evaluation strategies such as mouse tracking, the system usability scale, and a task-based user survey.

### 3.1 Evaluation methods

#### 3.1.1 Mouse tracking

Mouse tracking is used to identify the way the user interacts with the application by tracking the movements of the cursor (Freeman et al., 2011). This will help us in gaining data about how users navigate and interact with the visualization. This data will eventually help us in understanding the intuitiveness of the interface. In our scenario, it gives us information on how users navigate through the hierarchy, and the pathways they explore. We used the Tobii Pro Lab^6^ software to track the users' mouse movements.

#### 3.1.2 System usability scale

The System Usability Scale (SUS) is an evaluation approach (Brooke, 1995) consisting of 10 questions that help in evaluating the overall user experience and effectiveness of the system. This scale is widely adapted and proved to be a reliable tool for measuring a system's usability. It is a standard questionnaire consisting of a 5-point likert scale ranging from “Strongly Agree” to “Strongly Disagree” for each question.

#### 3.1.3 Task-based survey

Finally, a task-based user survey was used to measure the effectiveness of the system in enabling the user to accomplish specific tasks. These tasks were designed to imitate the real-world use cases that enable users to explore the hierarchies in different ways. Once the evaluation was completed, we gathered the data from the studies to analyze it for gaining further insights into the usability and effectiveness of the application. This study helped us in understanding the strengths and weaknesses of the system, which set a direction for further refinements of the system.

### 3.2 Evaluation setup

**Figure 13:**
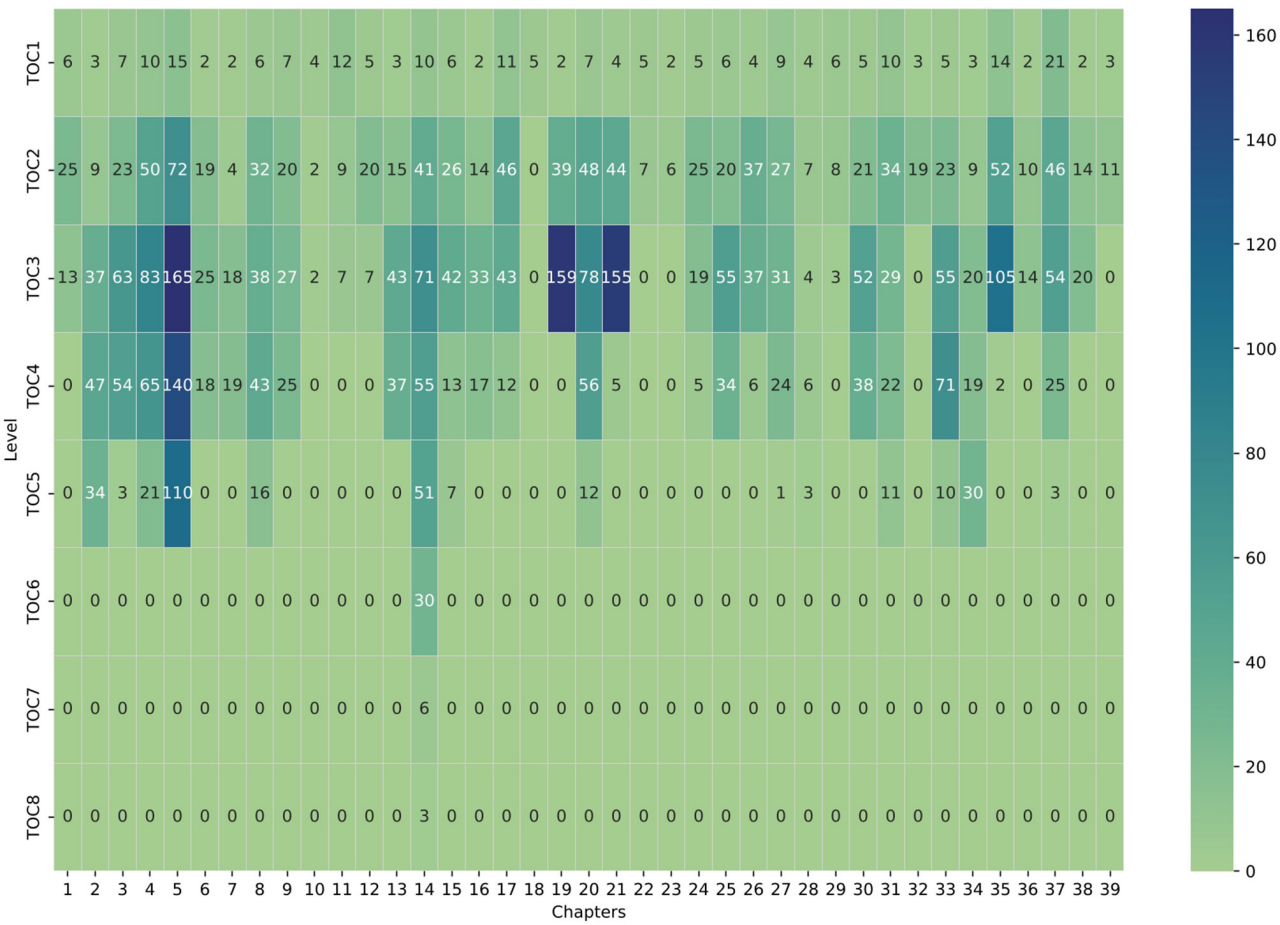
Section headings per chapter shown by Table of Contents (TOC) hierarchy levels. Darker blue denotes higher counts in a chapter's TOC hierarchy, while lighter green indicates lower counts.

Our dataset is sourced from a comprehensive rental law handbook (Harz et al., 2018), comprising 6 parts, 39 chapters, and a multi-tiered Table of Contents (TOC). Figure 13 illustrates the hierarchical distribution and depth, excluding keywords, context, and references from section content. The distribution plot reveals varied depth, mostly spanning levels 1 to 3, peaking at level 8 in a single instance. Notably, chapters 19 and 21 exhibit over 150 sections at level 3, underscoring the necessity for a tailored navigation solution to locate legal text references within these hierarchies.

**Figure 14:**
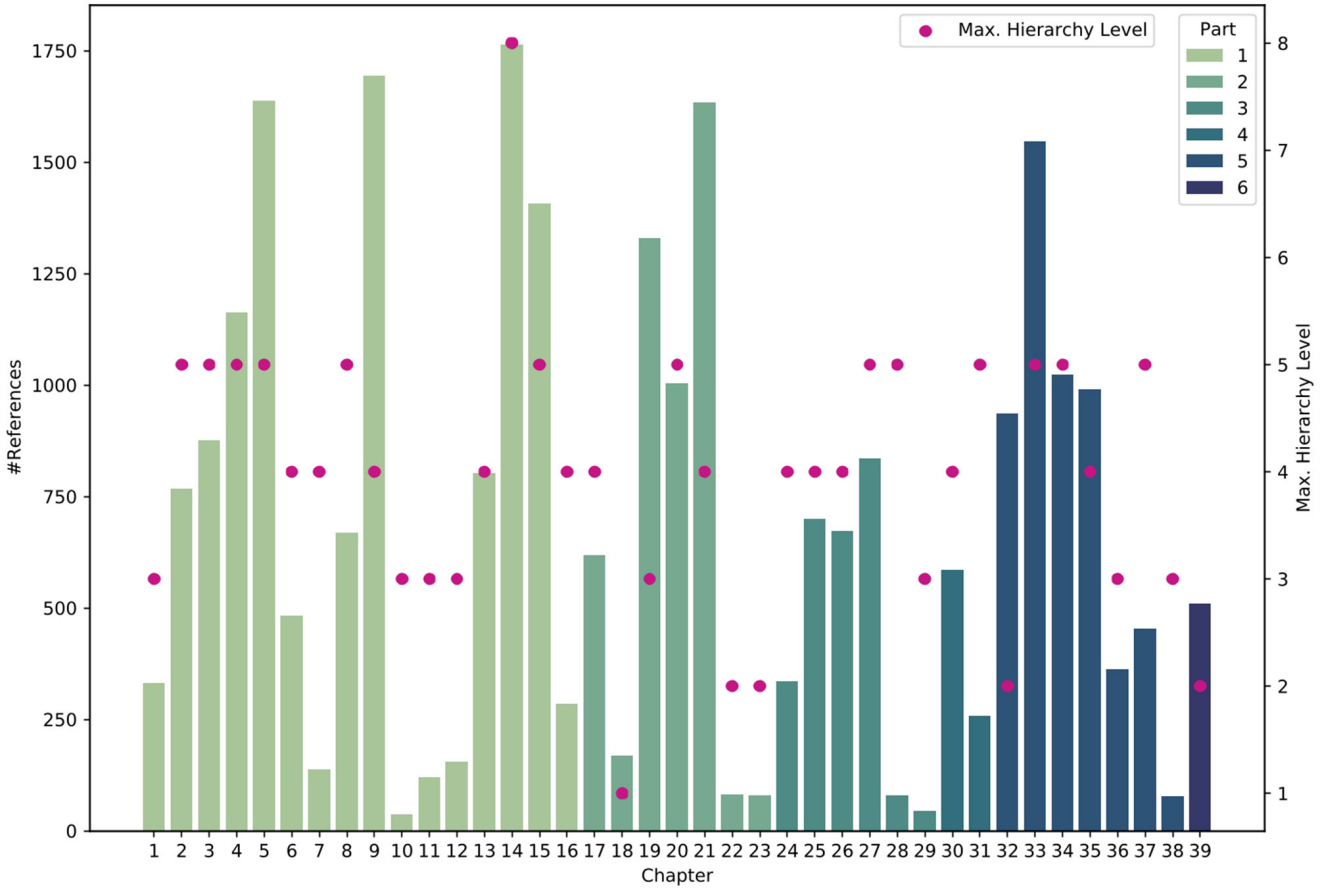
Statistics on the count of references and the maximum Table of Contents (TOC) level per chapter. The color indicates to which part of the textbook the respective chapter and its references belong.

Figure 14 visualizes which chapters belong to which part of the book, the maximum hierarchy level per chapter, and the number of references in each chapter. We can see that usually the maximum number of hierarchy levels varies between 2 and 5, while 8 levels were found once for chapter 14. The number of references varies substantially; in some chapters, we have less than 100, while in other chapters, there may be over 1,500. Note that these numbers indicate automatically extracted references. There are likely more references in each chapter, which were not captured by our specified extraction patterns. Manual examinations suggest that we currently extract about 90% of references in the given textbook.

For the usability tests, we have recruited a total of nine participants with legal backgrounds for the evaluation process. Thereof, we used two participants for a pilot study to confirm the test setup, and the results from these participants are omitted from our analysis. The study participants received a remuneration for their efforts (20 EUR). The evaluation was done in two stages:

- **Stage 1–Introduction and training:** We created readable and video material for introducing the participants to concept hierarchies and the visualization system we developed (covering the purpose and functionalities). Later, they were given time to familiarize themselves with the system. They were also informed that their mouse movements will be recorded and received a consent form to allow or prohibit the recording of additional audio and video footage.
- **Stage 2–Task-based user survey and SUS:** In this stage, the participants were given a set of tutorial tasks to be performed using the system with varying complexities. They had to fill out a part of the survey after each task. Finally, they were presented with an open task (which was the actual challenge after the tutorial) that allowed them to explore and navigate the system. This was done to understand how users perceive the system in terms of its intuitiveness. Once these tasks were performed, the participants filled in the usability survey (SUS).

The tutorial tasks were:

- **Task 1.1:**

- Select the top-down tab.
- Click on each part.
- Which part has the least number of chapters?

- **Task 1.2:**

- Select the part named “Teil 4 Miet- und wohnungseigentumsrechtliche Bezüge zum Offentlichen Recht einschließlich Steuerrecht.”
- Click on each chapter and compare which chapter has the least number of sections in the TOC 1 level.

- **Task 1.3:**

- Go back to the homepage.
- Select the part “Teil 1 Mietrecht.”
- Select chapter “Kapitel 10 Nebenpflichten.”
- Click on the “Preview” button of the third entry in TOC 1 and see how many references are shown.

- **Task 2.1:**

- Select the middle-out tab.
- Enter the word “Kündigung” in the search area.
- Find how many results of “Kündigung” are shown.
- Set the number of search results from 10 to 100. Go to the next page of results.

- **Task 2.2:**

- Select the middle-out tab.
- Enter the word “Kündigung” in the search area.
- Uncheck the box “TOC” for searching only in “Context.”
- Click on search and select the section “bbb) Kombination mit ordentlicher Kündigung” in the first result.
- Select “go to TOC.”
- Identify in which TOC level the section “bbb) Kombination mit ordentlicher Kündigung” is located.

- **Task 2.3:**

- Select the middle-out tab.
- Enter the word “Kündigung” in the search area.
- Uncheck the box “Context” for searching only in “TOC.”
- Click on search and select the topic “f) Kautionsverzug” in the first result.
- Select “go to chapter.”
- Identify in which TOC level you ended up.

- **Task 3.1:**

- Click on the middle-out tab.
- Enter the search term “§ 569 BGB” and click on search.
- Select the second result from the top.
- Click on the reference.
- Observe that the control moves to the bottom-up tab and shows the reference you clicked on as the new search term.

- **Task 3.2:** Is the reference you clicked on in Task 3.1 now also appearing in the result list of the bottom-up tab?
- **Task 3.3:** Find the number of times the reference is used.

The independent tasks were:

- **Task 4:** Find out how many times “§ 568 BGB” occurs and mention the chapters in which it occurs if they are different.
- **Task 5:** Find any verdict (German: Gerichtsurteil) in the current system and specify which one you found.

### 3.3 Data analysis

#### 3.3.1 User insights

When we analyzed the participants, we gathered some interesting insights as follows: According to our 7 responses, 5 users were in their law studies (passed the intermediate law exam) and 2 users have finished high school and started their bachelor's education.

Most of the users (5 out of 7) have reported that they did not work with hierarchical data so far. Hence, they were relatively new to the concept of hierarchical visualization, which helped us in understanding how the system is perceived by an inexperienced user.

When we explained to users our notion of hierarchical data and how it can be traversed using various approaches, almost all of them could quickly relate to the way they use this kind of traversal in day-to-day life (in our case going through textbooks). All users recognized that they have used top-down traversal before, 2 out of 7 already used the middle-out approach, and 1 person indicated familiarity with bottom-up traversal.

Even though users use textbooks in a digital format, most of the users (i.e., 4) still prefer physical textbooks over digital ones. When asked about how they typically use textbooks, all users reported that they follow more than one way of going through the textbook, based on the situation. We share the following responses regarding the users' interaction with a textbook:

- Scanning through the text to find a relevant concept or keyword (2 confirming responses).
- Using the index or table of contents to locate specific topics (4 confirming responses).
- Skimming the text to get a general sense of the content (2 confirming responses).
- Taking notes or highlighting important concepts (3 confirming responses).
- Combination of all of the above (4 confirming responses).

Most of the users go through case books (4 users) compared to textbooks (3 users) for their learning needs. Case books delve into specific cases to illustrate legal principles in action, while legal textbooks offer a broader understanding of legal concepts, theories, and doctrines without focusing extensively on individual cases. Both are essential for legal education, providing different perspectives and depth of knowledge. Follow-up studies for concept hierarchy visualization in legal education settings shall also consider case books in addition to textbooks. Overall, working with hierarchies in the top-down manner seemed the most familiar approach to the study participants.

#### 3.3.2 User survey insights

As part of a task-based survey carried out to understand how participants perceive the system we developed, we created a set of tasks that helps us in understanding how users react when users are presented with a system they are not familiar with. Following are the insights we gathered from the survey.

Users were able to complete 80% of the practical tasks. Even though most of the users were only familiar with the top-down approach, they were able to finish tasks based on middle-out and bottom-up approaches, too. However, we noticed higher success rates in task completion in the top-down approach and the lowest success rate in the bottom-up approach on average.

Users were able to complete 100% of the exploratory tasks that were provided. They quickly could identify the right approach to choose for finishing the task and had varied approaches with the majority of them going for top-down (43%) or middle-out (43%) approaches. We also observed that some users who used the middle-out approach for doing independent tasks were only familiar with the top-down approach before using the system. This can indicate the intuitiveness of the system. After finishing the tasks, 43% of the users were in favor of using the middle-out approach over other approaches followed by the bottom-up approach (29%) and top-down approach (28%).

We also observed that 2 participants found it difficult to work with the bottom-up approach and found it to be difficult in transitioning to the bottom-up approach from the middle-out approach. When it comes to understanding the visualization and the way the data were presented, we noticed that 43% of the users found it neutral, while 28% found it easy and other 28% found it to be difficult.

We also found out that the majority of the people who prefer physical textbooks over digital textbooks found this tool to be helpful and would like to use it in future. Participants were asked to compare the current system with other tools they often use for referring case laws and other materials. These alternative tools are Beck online^7^ and Beck E-Library^8^. We had the following responses:

- Our designed prototype (3 votes) is perceived to be equal to Beck E-library (3 votes) when discovering a new topic in terms of creating a bigger picture by showing all information in one place.
- The proposed system performs better (4 votes) than Beck online (3 votes) and Beck E-library (2 votes) when users want to review or gather more information about a known topic.
- The proposed system (3 votes) is rated as equal to Beck online (3 votes) for research purposes.

#### 3.3.3 SUS-based insights

We have used SUS (System Usability Scale) (Brooke, 1995) to evaluate the usability of the current system as seen by the potential users and the following are our observations.

The proposed system was rated with a SUS of 62.5% on average across all participants, where the highest score was 80% and the lowest score was 45%. We noticed that these scores also reflect the number of tasks participants were able to complete correctly. We noticed that a lower SUS was mainly attributed to unclear back navigation to the home page, and bottom-up hierarchy interactions.

#### 3.3.4 Mouse tracking insights

**Figure 15:**
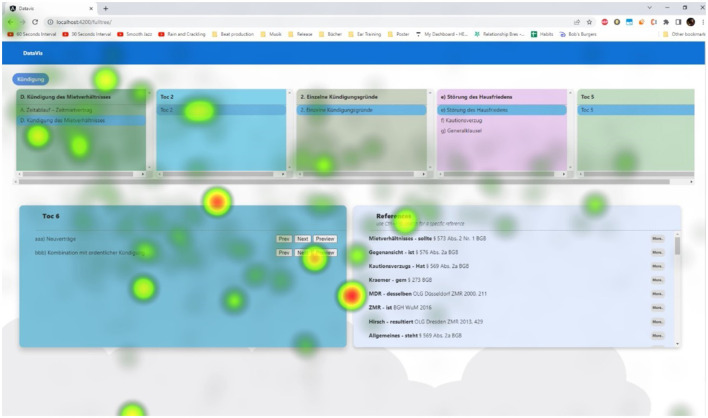
Heatmap from participants using the mouse in the “TOC view” exploring the hierarchy.

We tracked participants' mouse movements during independent tasks to understand their interactions with the application and identify frequently used elements. Heatmaps revealed key areas of interest. Notably:

- Users preferred the browser back button over the system's, finding it more intuitive, a sentiment echoed in their feedback (see Figure 15).
- The “Preview” button was heavily used for reference filtering in the “TOC view” (see Figure 15).
- Bottom-up and middle-out approaches saw limited use of the TOC view.
- In middle-out, search, references, context, and filter were the most used elements, indicating the effectiveness of placing reference and context tools here.
- Interestingly, bottom-up and middle-out visualizations showed similar heatmap patterns.

Participants showed interest in understanding reference context, using tools as aids for reviewing familiar topics. They often rested the mouse while browsing information. While mouse tracking supported assumptions and feedback, it lacked insight into focused elements during resting periods. Gaze tracking could enrich this understanding. We plan to repeat the study using eye-tracking software for deeper insights into user interests.

## 4 Discussion

### 4.1 Further possible applications

This study focuses on designing an interactive system for legal education. However, the hierarchical visualization of textbooks could benefit various domains. For instance, in humanities, researchers often analyze textbook passages for specific mentions of gender identities, religious groups, or historical figures. The middle-out and bottom-up traversal's support for searching occurrences of these terms could greatly aid in this research, forming a basis for detailed analysis. Similarly, in the medical field, extracting terms such as symptoms or therapy options from textbooks could assist medical professionals in their work.

The extraction and visualization of textbook knowledge have promising applications, especially within the realm of Large Language Models (LLMs). Trust issues often surround LLMs, notably in critical fields such as medicine and law, where misinformation carries high stakes. To bolster their reliability, training LLMs with high-quality textbook input can enhance the accuracy of their output (Gunasekar et al., 2023).

To further fortify trust and clarity in LLM-generated claims, extracting evidence from their training data or external reliable sources becomes pivotal. Wehnert termed this approach “Justifiable Artificial Intelligence,” emphasizing the need for evidence-backed outputs (Wehnert, 2023). Leveraging concept hierarchies or knowledge graphs via a retriever module (Wehnert et al., 2019b) expedites evidence retrieval compared to scanning entire textbooks used in model training.

Ultimately, employing candidate ranking and visualization techniques such as middle-out or bottom-up facilitates users in accessing and comprehending potential evidence before delving into the full text of selected sources.

LLMs exhibit proficiency in extracting common themes from legal facts, as observed in the study by Drápal et al. (2023). Their study highlights the LLMs' adeptness at discerning themes, often aligning closely with human-derived themes, albeit differing slightly in abstraction levels.

Similar success emerges in various domains, such as using LLMs in educational physics engineering contexts for tasks such as “exam wrappers” (Gamieldien et al., 2023). Furthermore, these models excel in extracting intricate structures, such as legal pathway delineation (Janatian et al., 2023) and hierarchical concept relations in materials chemistry from scientific texts (Dunn et al., 2022).

Drápal et al. (2023)'s theme extraction approach might extend to hierarchical theme extraction, potentially visualized using our prototype and explored using the top-down traversal.

### 4.2 Conclusion

In conclusion, our prototype demonstrates promise in assisting users with interacting and comprehending concept hierarchies, developed through a model-based human-centered design approach. We propose an intuitive and engaging tool.

Following this approach, we assessed usability and intuitiveness through methods such as SUS for systematic usability evaluation, task-based surveys, and mouse tracking to observe user interactions within the system.

User feedback indicates a positive perception of the tool. The statement “The tool is good being in the 1st iteration” suggests users see potential for further development. It also opens the door to a new level of interactive learning, resulting in a more engaging and efficient process to consume textbook information with entities of interest (such as the references in this case). This could also be considered a preliminary step toward the ever-developing field of data visualization. We also compiled a list of strengths and limitations for the visualization provided by participants.

### 4.3 Strengths and limitations

Consolidating all the information we gathered from our study, the following are the strengths and weaknesses of the system as seen by users.

**Strengths:**

- The visualization is easy to use overall, users can confidently use the visualization with the help of some documentation/user guide.
- The visualization provides better flexibility in searching for concepts when compared to Beck Online and Beck E-Library.
- The visualization provides better search and filtering options compared to Beck Online as commented by participants.
- This visualization is suitable for textual hierarchical data.
- This visualization is meant to be for a web-based system, thus it can be accessed anywhere.
- This visualization is useful for quickly going through references and concepts.

**Limitations:**

- The visualization is still in the prototype phase and has not fully matured yet (e.g., visual design).
- Some of the features are unclear (users perceive the middle-out and bottom-up approaches to be the same).
- We need a better navigation functionality between the visualizations (moving between the middle-out and bottom-up approaches by clicking on the reference has been confusing for users at times).
- There is a need to show hints and comments about how to effectively use the system.
- The system gives a better overview of previous levels by displaying all the nodes from each level on top of the screen with the highlighted path, but no overview is available for the lower levels in the TOC view of all three approaches.

### 4.4 Future work

We will use the evaluation feedback to guide our future steps:

**Streamlined navigation:** Addressing user concerns, notably regarding backward navigation, aiming for a more intuitive flow.

**Complete hierarchy display:** Exploring methods to showcase the entire hierarchy in a side panel, similar to Windows Explorer's layout.

**Subscription feature:** Considering a subscription system for users to track specific hierarchy nodes, facilitating notifications upon related database modifications or additions. This can also be used for querying further data sources, such as parliament speeches about legal issues (Bönisch et al., 2023). This expands the system's role from exploration to recommendation.

**Enhanced search functionality:** Improving the search system with live links and suggested searches to enhance usability.

**Detailed metadata:** Intending to offer comprehensive metadata (book source, author, publisher, and citations) aligning with user interests.

**Expanded data sources:** Enriching the database by incorporating case law, articles, and laws.

**Hierarchy editing tools:** Providing users with tools to compare, modify, or report inaccuracies in imported hierarchies.

**Integrating middle-out and bottom-up approaches:** Exploring methods to merge middle-out and bottom-up views, potentially enabling specific searches within references in the middle-out view to encompass bottom-up functionalities.

## Data availability statement

The raw data supporting the conclusions of this article will be made available by the authors, without undue reservation.

## Ethics statement

Ethical approval was not required for the studies involving humans because we performed behavioral research to determine the usability of an interactive system. The studies were conducted in accordance with the local legislation and institutional requirements. The participants provided their written informed consent to participate in this study. Written informed consent was obtained from the individual(s) for the publication of any potentially identifiable images or data included in this article.

## Author contributions

SW: Conceptualization, Data curation, Funding acquisition, Methodology, Project administration, Validation, Visualization, Writing—review & editing. PC: Conceptualization, Formal analysis, Investigation, Methodology, Software, Visualization, Writing—original draft. JA: Conceptualization, Investigation, Methodology, Writing—review & editing. ED: Resources, Supervision, Writing—review & editing.

## Footnotes

^1^Link to the Code repository: https://github.com/pranee525/Datavis.

^2^ https://developers.google.com/chart/interactive/docs/gallery/wordtree

^3^Guide for setting up angular project: https://angular.io/guide/setup-local.

^4^Guide for setting up Material UI: https://material.angular.io/guide/getting-started.

^5^ http://orientdb.org/

^6^Tobii Pro Lab: https://www.tobii.com/products/software/behavior-research-software/tobii-pro-lab.

^7^ https://beck-online.beck.de/Home

^8^ https://www.beck-elibrary.de/
