# Supplementary material for: A dynamic approach for visualizing and exploring concept hierarchies from textbooks
Source: Front Artif Intell. 2024 Feb 8;7:1285026. doi: 10.3389/frai.2024.1285026 (PMC10881833; doi:10.3389/frai.2024.1285026)
Supplement: Supplementary file 1 [file Data_Sheet_1.pdf]

Supplementary Material of the User Study

1 SURVEYS AND THEIR RESULTS

Questions	Options
Technical Background (Education level)	High School (Completed)<br>High School (Not Completed)<br>Graduate (e.g., Bachelor's, 1st Degree)<br>Post Graduate (e.g., Master's, State exam, 2nd Degree)<br>Professional Degree<br>No Schooling Completed<br>Doctorate Degree<br>Professorship<br>Zwischenprüfung
1. Have you ever worked with hierarchical data?	Yes<br>No
2. Please rate your level of familiarity with exploring concept hierarchies.	No Familiarity<br>Beginer<br>Intermediate<br>Expert

Table S1. Participant Background Part 1

Questions	Options
3. Which of the following techniques have you used before to explore concept hierarchies?	-Top-Down (from the root of the hierarchy down to the most detailed leaf nodes)<br>-Middle-Out (starting from one location inside the hierarchy and moving up or down)<br>-Bottom-Up (from the bottom leaf nodes of a hierarchy towards the root)
4. How do you typically interact with textbooks when you want to find out more about a specific topic? (Select all that apply)	-Scanning through the text to find a relevant concept or keyword (searching for a specific term)<br>-Using the index or table of contents to locate specific topics<br>-Skimming the text to get a general sense of the content (understand the structure and how content is presented)<br>-Taking notes or highlighting important concepts<br>-Combination of all of the above
7. What do you use the most for learning?	Textbook<br>Case Book<br>Lecture
8. Which field of Law interests you the most?	Public Law<br>Civil Law<br>Criminal Law
9. How much knowledge do you have about tenancy law (Mietrecht)?	No Familiarity<br>Beginner<br>Intermediate<br>Expert
10. How often do you use textbooks in physical format (printed version) ?	Scale: 1-5
11. How often do you use textbooks in digital format (e-book, pdf)?	Scale: 1-5
12. Do you prefer a physical or a digital textbook?	Physical Textbook<br>Digital Textbook

Table S2. Participant Background Part 2

**Questions**	**Options**
Task 1.1<br>- Select the top-down tab<br>- Click on each part<br>- Which part has the least number of chapters?	Open Answer
Task 1.2<br>- Select the part named ”Teil 4 Miet- und wohnungseigentumsrechtliche Bezüge zum Offentlichen Recht einschließlich Steuerrecht”<br>- Click on each chapter and compare which chapter has the least number of sections in the TOC 1 level	Open Answer
Task 1.3<br>- Go back to the homepage<br>- Select the part ”Teil1 Mietrecht”<br>- Select chapter ”Kapitel 10 Nebenpflichten”<br>- Click on the “Preview” button of the third entry in TOC 1 and see how many references are shown	10<br>5<br>3
Task 2.1<br>- Select the middle-out tab<br>- Enter the word ”Kündigung” in the search area.<br>- Find how many results of ”Kündigung” are shown.<br>- Set the number of search results from 10 to 100.<br>- Go to the next page of results	Open Answer
Task 2.2<br>- Select the middle-out tab<br>- Enter the word ”Kündigung” in the search area.<br>- Uncheck the box ”TOC” for searching only in ”Context”<br>- Click on search and select the section ”bbb) Kombination mit ordentlicher Kündigung” in the first result<br>- Select ”go to TOC”<br>- Identify in which TOC level the section ”bbb)) Kombination mit ordentlicher Kündigung ” is located	Open Answer

Table S3. Task List Part 1

**Questions**	**Options**
Task 2.3<br>- Select the middle-out tab<br>- Enter the word ”Kündigung” in the search area.<br>- Uncheck the box ”Context” for searching only in ”TOC”<br>- Click on search and select the topic ”f) Kautionsverzug” in the first result<br>- Select ”go to chapter”<br>- Identify in which TOC level did you end up	Open Answer
Task 3.1<br>- Click on the middle-out tab<br>- Enter the search term ”§ 569 BGB” and click on search.<br>- Select the second result from the top.<br>- Click on the reference<br>- Observe that the control moves to the bottom-up tab and shows the reference you clicked on as the new search term	Open Answer
Task 3.2 Is the reference you clicked on in Task 3.1 now also appearing in the result list of the bottom-up tab?	Open Answer
Task 3.3 Find the number of times the reference is used.	Open Answer
Task 4 Find out how many times ”§ 568 BGB” occurs and mention the chapters in which it occurs if they are different.	Open Answer
Task 5 Find any verdict (Gerichtsurteil) in the current system and specify which one you found.	Open Answer
Task 6<br>- Select Middle-out tab<br>- Enter the search term ”Kündigung” and click on search<br>- Now find the “filter chapters” option and select ”Kapitel 16 Pacht”<br>- Did the search results change?	Open Answer
Which of the three available approaches did you use for the Task 5?	Top-Down<br>Middle-Out<br>Bottom-Up

Table S4. Task List Part 2

Questions	Options
How easy was it for you to understand the visualization presented?	Scale: 1-5
Which approach did you find most effective in helping you solve the independent task (last task)?	Top-Down<br>Middle-Out<br>Bottom-Up
With which approach were you able to locate specific topics within the visualization easily?	Top-Down<br>Middle-Out<br>Bottom-Up
How do you rate the visual presentation of hierarchy on terms of understanding and clarity?	Very Clear<br>Clear<br>Neutral<br>Unclear<br>Confused
Were there any parts of the hierarchy visualization that were confusing or difficult to understand?	Open Answer
If you have to go through a textbook for some topic then how likely are you going to use this tool to support your search?	Scale: 1-5
Do you have any additional comments that are not covered in the previous questions, feel free to let us know.	Open Answer
Compare the application you have used now to Beck Online and Beck E-Library explain briefly how you feel about this system.	Open Answer
Which application would you use for the following tasks<br>A. Reading about a topic for the first time<br>B. Reviewing an already known topic<br>C. Research for an academic paper	Current System<br>Beck Online<br>Beck E-Library<br>Other
In case you selected the option ”Other” in the previous question, can you specify which system do you use?	Open Answer

Table S5. Post Questionnaire

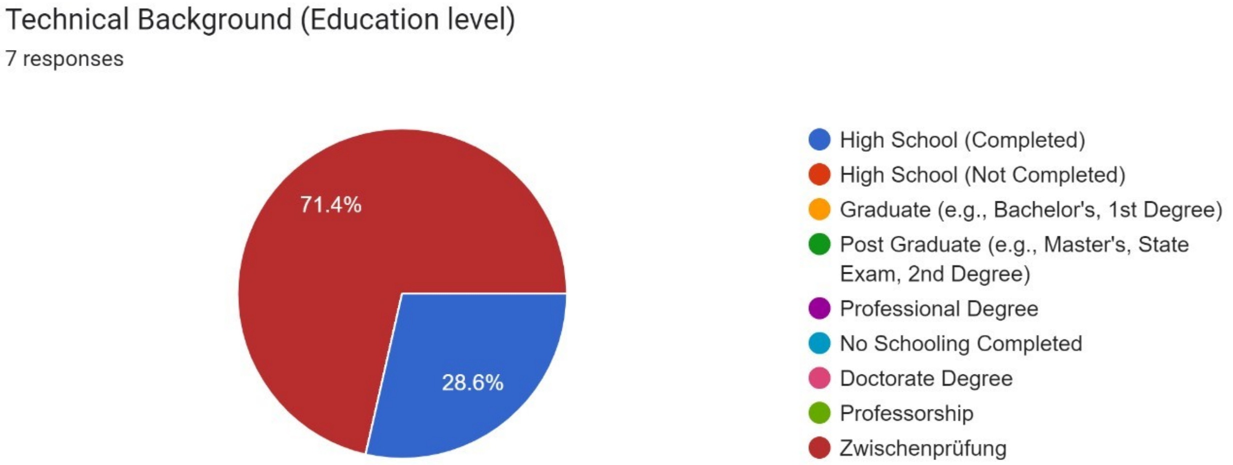

Figure S1. Technical Background

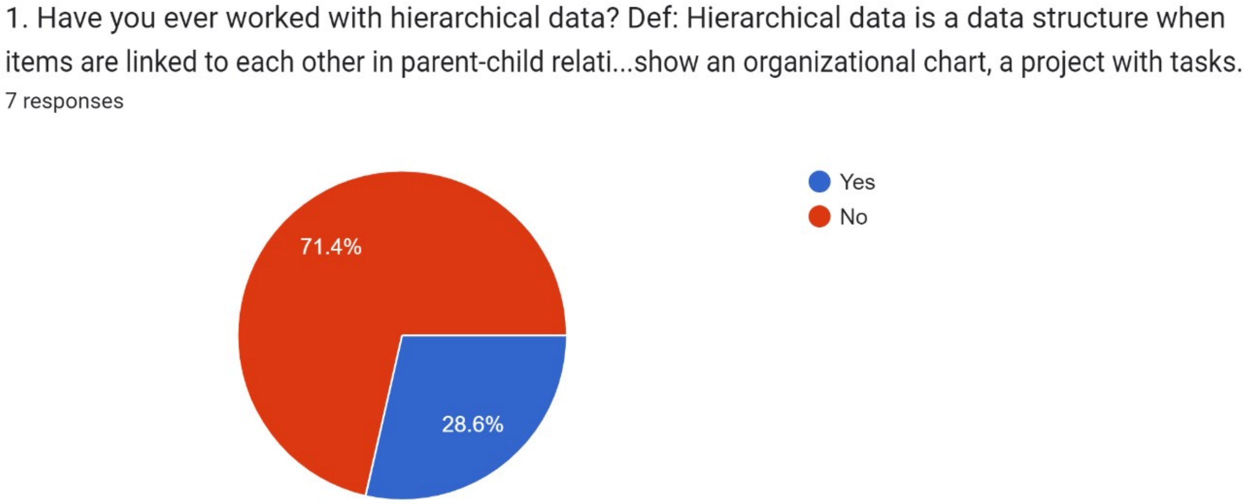

Figure S2. Users' knowledge of hierarchical data

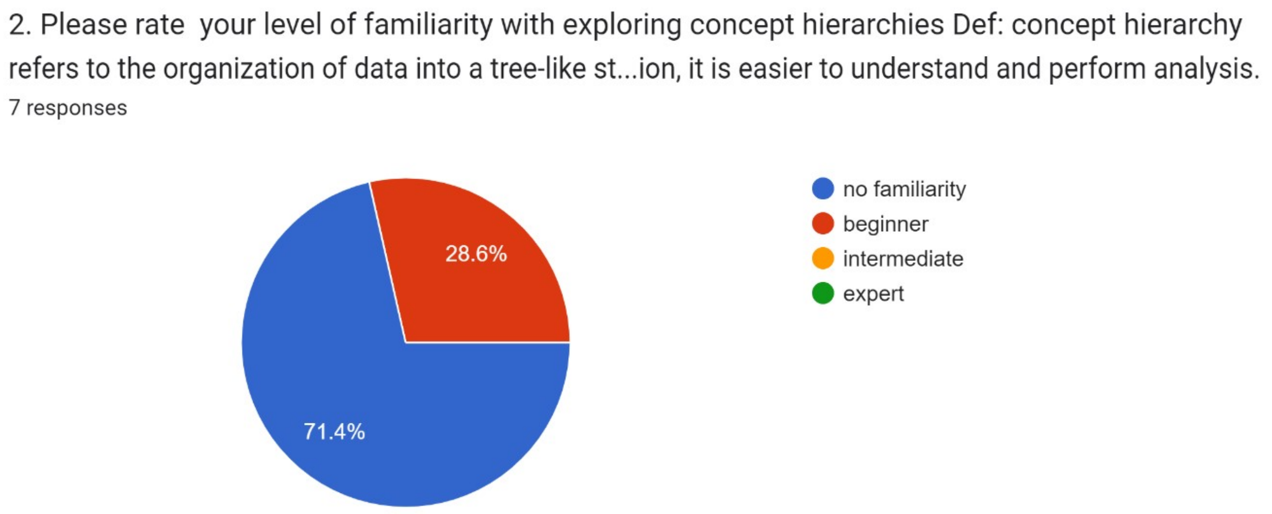

Figure S3. Users' familiarity with hierarchical data

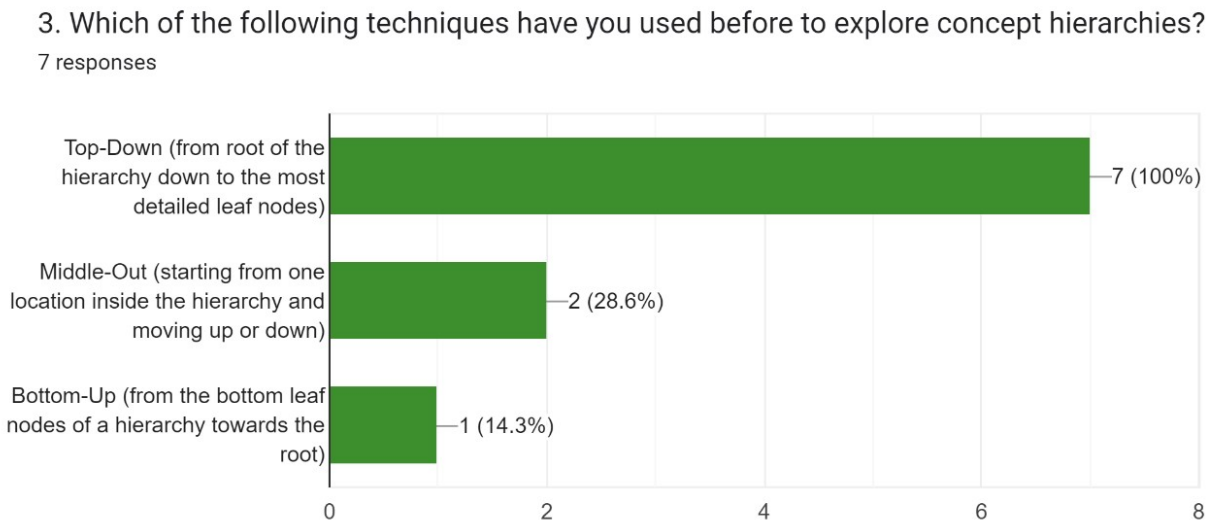

Figure S4. Techniques Users are familiar with

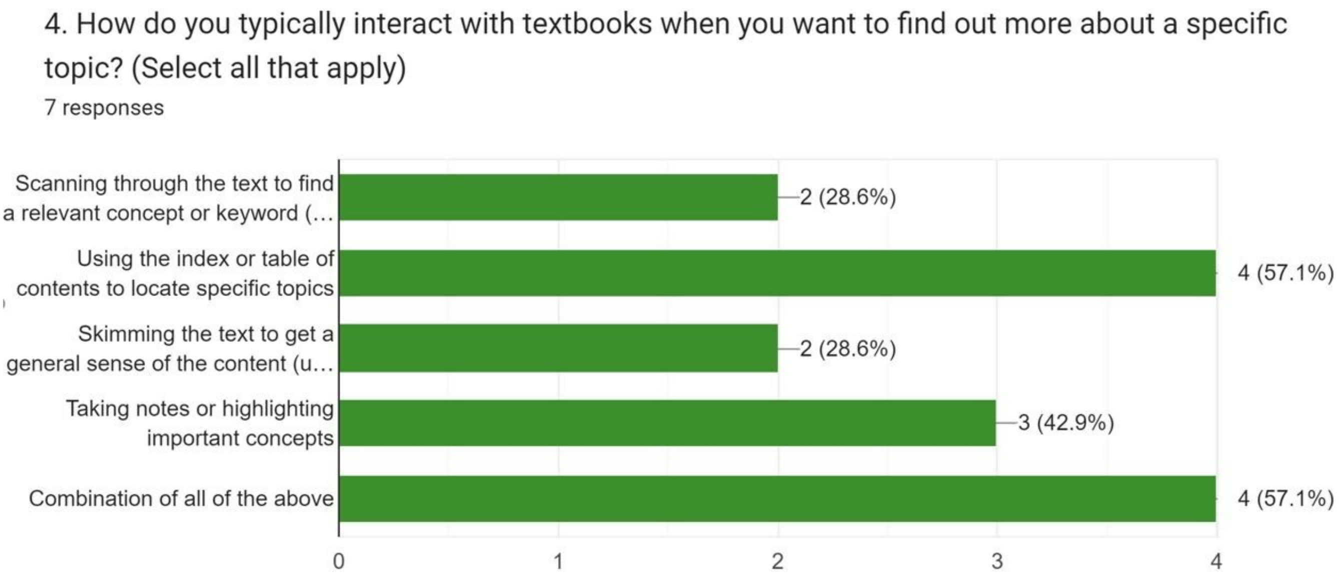

Figure S5. User's way of interaction with textbooks

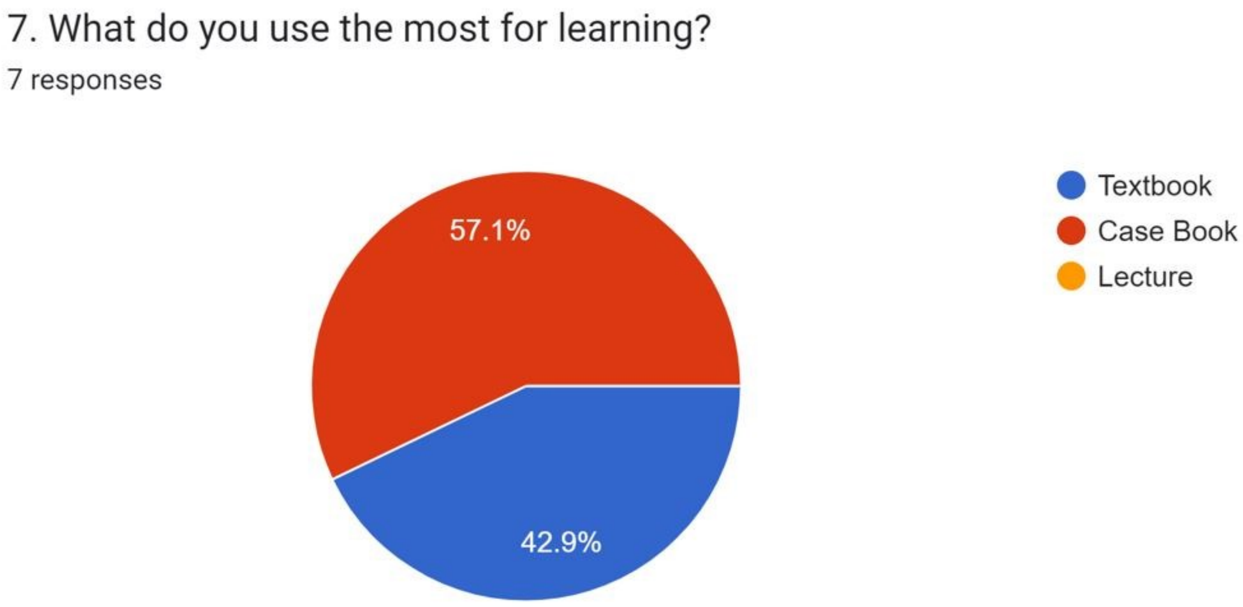

Figure S6. Users's most used learning material

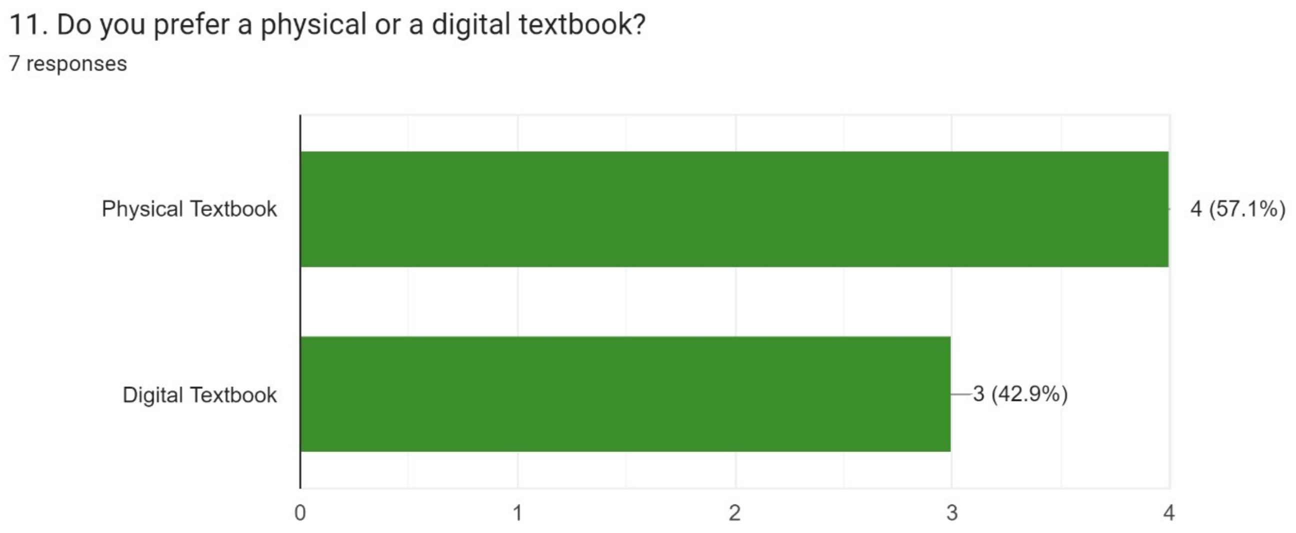

Figure S7. Users' preferred mode for reading textbooks

Statement	Strongly Disagree	Disagree	Neutral	Agree	Strongly Agree
I think that I would like to use this system frequently.	1	2	3	4	5
I found the system unnecessarily complex.	1	2	3	4	5
I thought the system was easy to use.	1	2	3	4	5
I think that I would need the support of a technical person to be able to use this system.	1	2	3	4	5
I found the various functions in this system were well integrated.	1	2	3	4	5
I thought there was too much inconsistency in this system.	1	2	3	4	5
I would imagine that most people would learn to use this system very quickly.	1	2	3	4	5
I found the system very cumbersome to use.	1	2	3	4	5
I felt very confident using the system.	1	2	3	4	5
I needed to learn a lot of things before I could get going with this system.	1	2	3	4	5

Table S6. System Usability Scale (SUS)

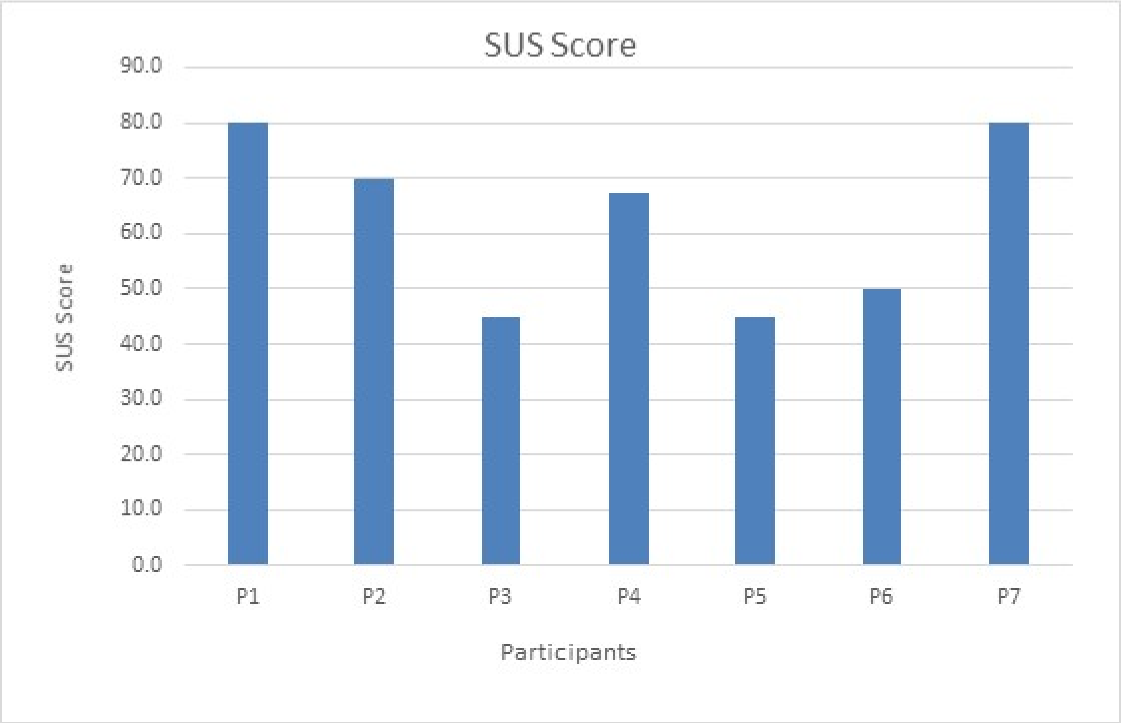

Figure S8. Users' System Usability Score (SUS)

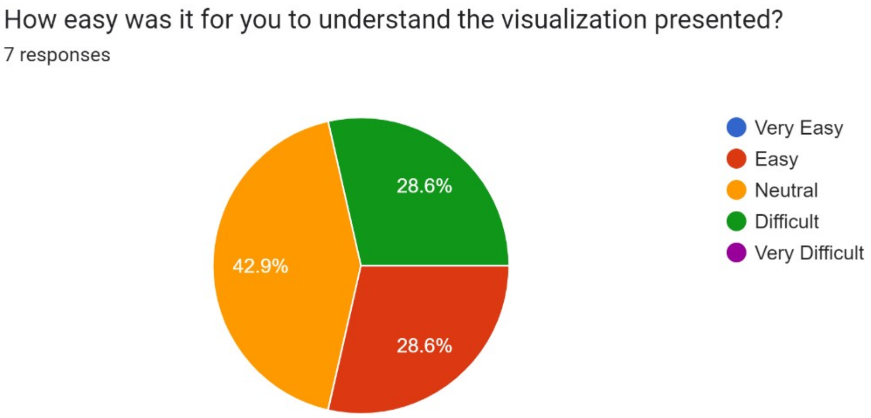

Figure S9. Users' View on Difficulty of Current System

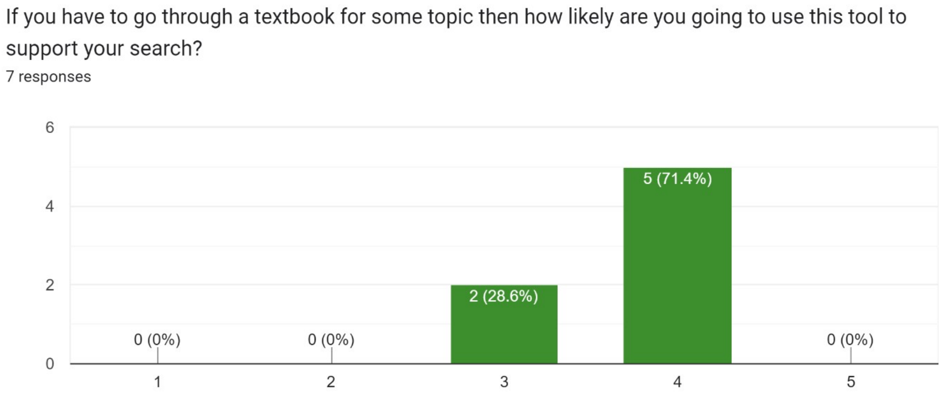

Figure S10. Users' Support

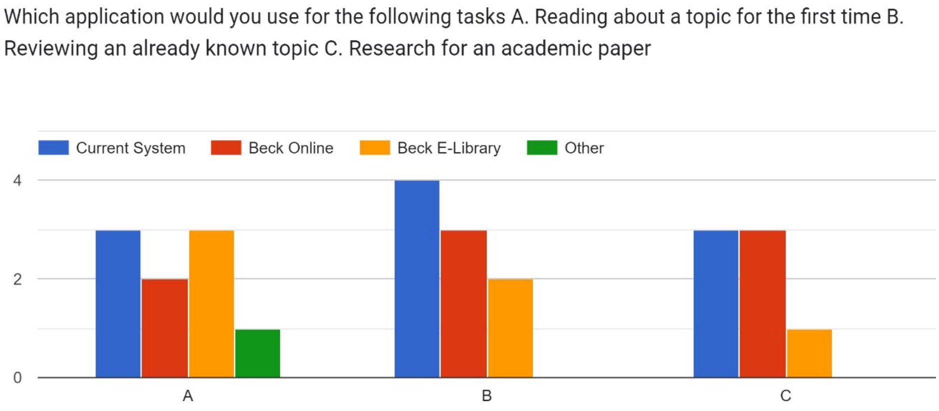

Figure S11. Users' Choice of Using the Current System

Participant Id	Education level	Worked with hierarchical data?	Familiarity with concept hierarchies
P1	Zwischenprüfung	No	no familiarity
P2	Zwischenprüfung	No	no familiarity
P3	High School (Completed)	Yes	beginner
P4	Zwischenprüfung	Yes	beginner
P5	Zwischenprüfung	No	no familiarity
P6	Zwischenprüfung	No	no familiarity
P7	High School (Completed)	No	no familiarity

Table S7. Participant Background

Participant Id	Techniques to explore concept hierarchies	Textbook Interaction Method	Learning Material
P1	Top-Down	Using the index or table of contents, Skimming the text, Taking notes or highlighting important concepts	Case Book
P2	Top-Down	Scanning through the text, Using the index or table of contents	Case Book
P3	Top-Down, Middle-Out, Bottom-Up	Using the index or table of contents, Taking notes or highlighting important concepts	Case Book
P4	Top-Down, Middle-Out	Scanning through the text, Using the index or table of contents, Skimming the text, Taking notes or highlighting important concepts, Combination of all of the above	Textbook
P5	Top-Down	Combination of all of the above	Case Book
P6	Top-Down	Combination of all of the above	Textbook
P7	Top-Down	Combination of all of the above	Textbook

Table S8. Participant interaction with textbooks

Participant Id	Field of Law	Knowledge on tenancy law	Use of physical textbooks	Use of digital textbooks	Textbook preference
P1	Criminal Law	beginner	4	1	Physical Textbook
P2	Civil Law	beginner	3	4	Digital Textbook
P3	Civil Law	no familiarity	4	3	Physical Textbook
P4	Public Law	beginner	4	2	Physical Textbook
P5	Civil Law	beginner	5	3	Physical Textbook
P6	Civil Law	no familiarity	2	4	Digital Textbook
P7	Criminal Law	beginner	3	5	Digital Textbook

Table S9. Participant Interests 2

Participant	Task 1.1	Task 1.2	Task 1.3	Task 2.1	Task 2.2
P1	Teil 6	Kapitel 31	6	201	Toc 6
P2	Teil 6	TOC 1	6		TOC 6
P3	6	30	6		TOC 6
P4	teil 6	kapitel 30	36	2720	6
P5	Teil 6	Kapitel 30	6		TOC 6
P6	Teil 6	Kapitel 31 Steuerrecht	6		Toc 6
P7	Teil 6	Ver Verwaltungsrecht	6	2720	TOC 6

Table S10. Participant Task Results 1

Participant	Task 2.3	Task 3.1	Task 3.2	Task 3.3	Task 4
P1	Toc 1	yes	yes	1	3, 8, 14, 34
P2	TOC 1	yes	Ja	1	14, 34, 14, 8, 3
P3	TOC1	yes	Yes		4
P4	1	yes			24
P5	TOC 1	yes	yes		
P6	Toc 1	yes	ja		
P7	TOC 1	yes	Yes		6

Table S11. Participant Task Results 2

Participant	Task 5	Approach for Task 5	Understanding of Visualization	Most Effective Approach
P1	OLG Schleswig NJW 1995,2859	Top-Down	Easy	Top-Down Approach
P2	OLG Hamm ZMR 1988, 138	Top-Down	Easy	Top-Down Approach
P3	OLG Schleswig NJW 1995, 2859	Bottom-Up	Difficult	Bottom-Up Approach
P4	olg schleswig NJW 1995, 2859	Middle -Out	Neutral	Middle-Out Approach
P5	BGH (NJW 1991)	Middle -Out	Neutral	Middle-Out Approach
P6			Difficult	Top-Down Approach
P7	toc 6	Bottom-UP	neutral	Middle-Out Approach

Table S12. Participant Task Results 3

Participant	Topic Location Approach	Hierarchy Clarity	Confusing Parts
P1	Middle-Out Approach	Clear	
P2	Top-Down Approach, Bottom -Up Approach	Clear	task 3.3
P3	Middle-Out Approach	Neutral	yes, the middle-out approach
P4	Top-Down Approach	Clear	
P5	Top-Down Approach	Neutral	yes
P6	Middle-Out Approach	Unclear	3.1, 3.2, 3.3
P7	Bottom -Up Approach	Clear	No

Table S13. Participant Suggestions

Participant	Would Like to Use Frequently	System Complexity	Easy to Use	Need Technical Support	Well Integrated Functions
P1	3	2	4	1	4
P2	4	2	3	2	3
P3	3	2	2	4	4
P4	4	3	4	2	3
P5	3	2	2	3	3
P6	2	2	3	4	4
P7	4	2	4	1	4

Table S14. Participant SUS Feedback 1-5

Participant	Inconsistency in System	Easy to Learn	Hard to Use	Confidence in Using	Learning Needed
P1	1	5	1	3	2
P2	2	3	1	4	2
P3	5	2	3	2	1
P4	2	4	2	3	2
P5	2	4	4	1	4
P6	4	4	2	2	3
P7	1	4	2	4	2

Table S15. Participant SUS Feedback 6-10

2 HEATMAPS

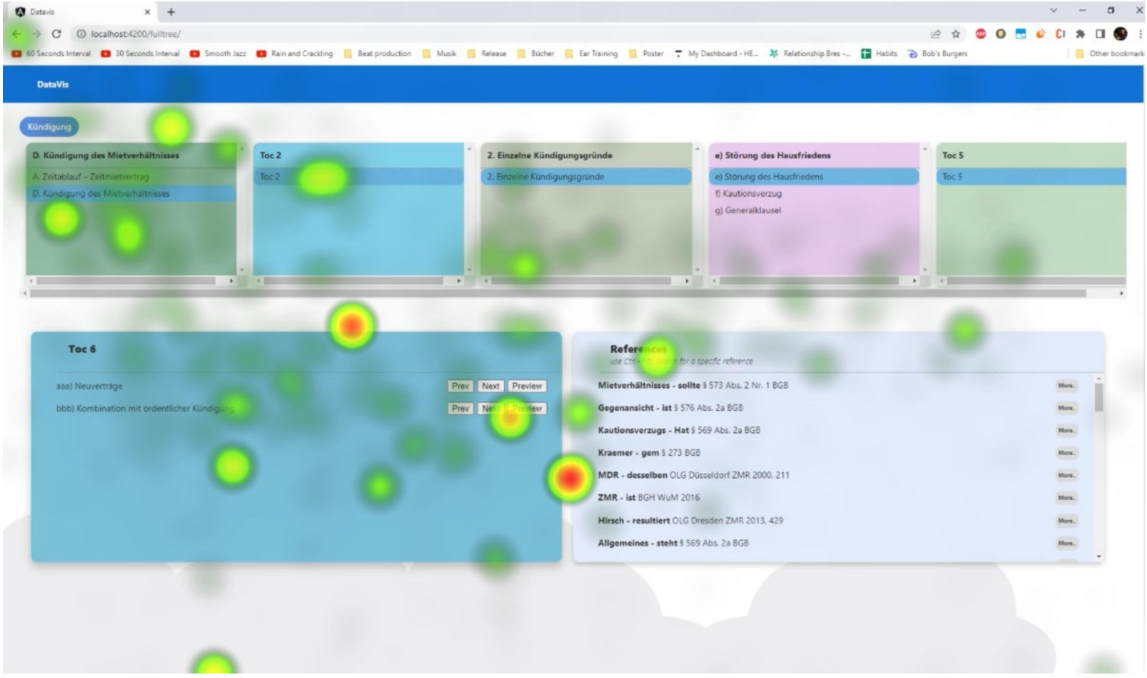

Figure S12. Heatmap from participants using the mouse in the 'TOC View' exploring the hierarchy

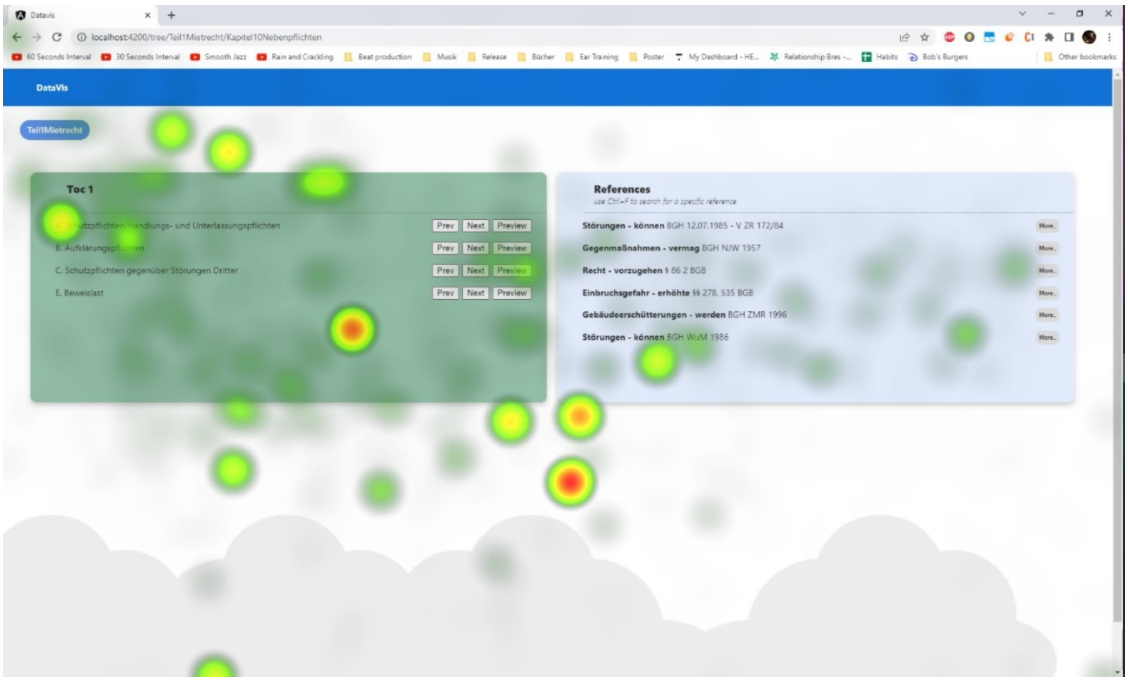

Figure S13. Heatmap from participants using the mouse in the ‘TOC View’

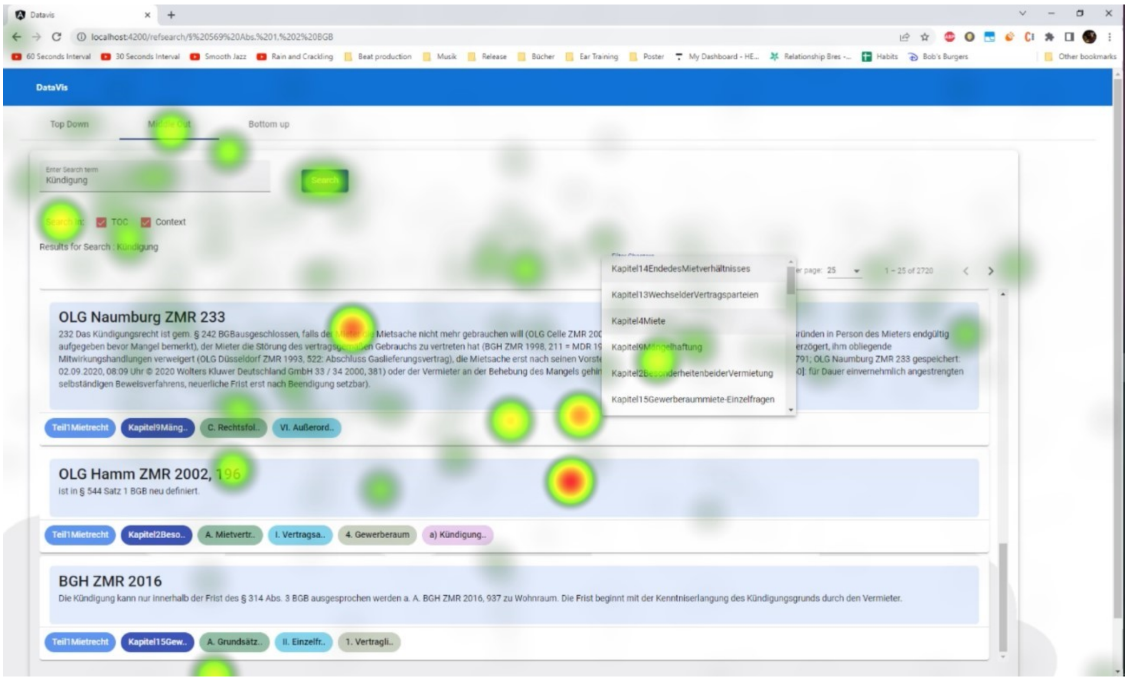

Figure S14. Heatmap from participants in the ‘Middle-Out’ approach exploring the hierarchy

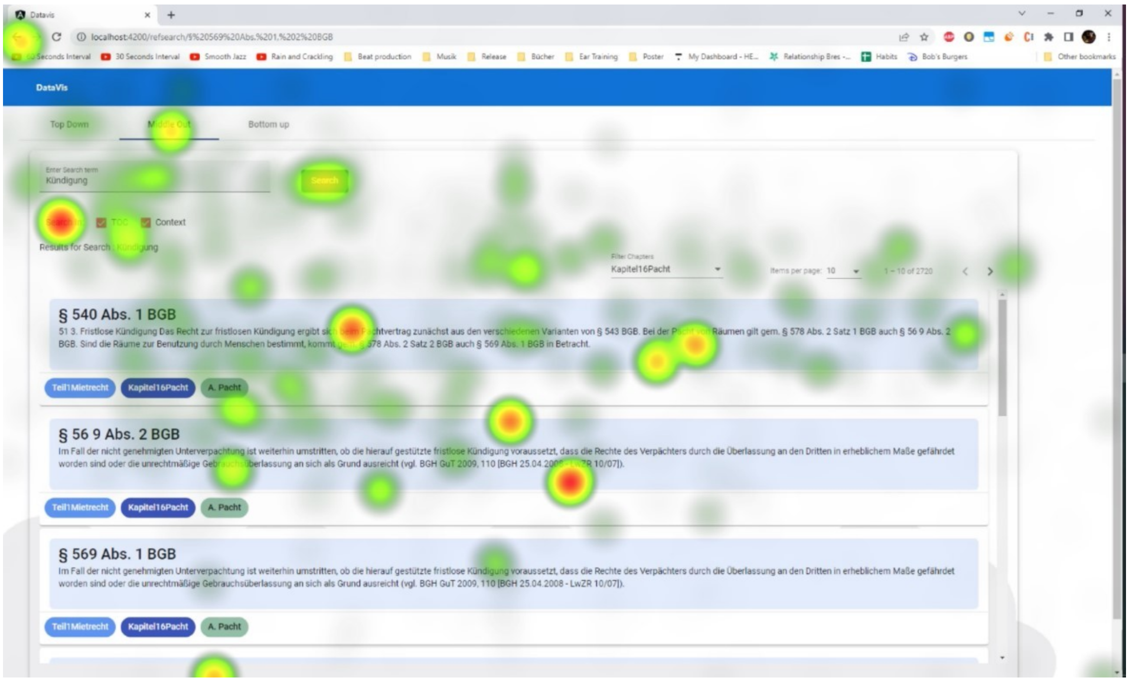

Figure S15. Heatmap from participants using the mouse in the 'Middle-Out' approach

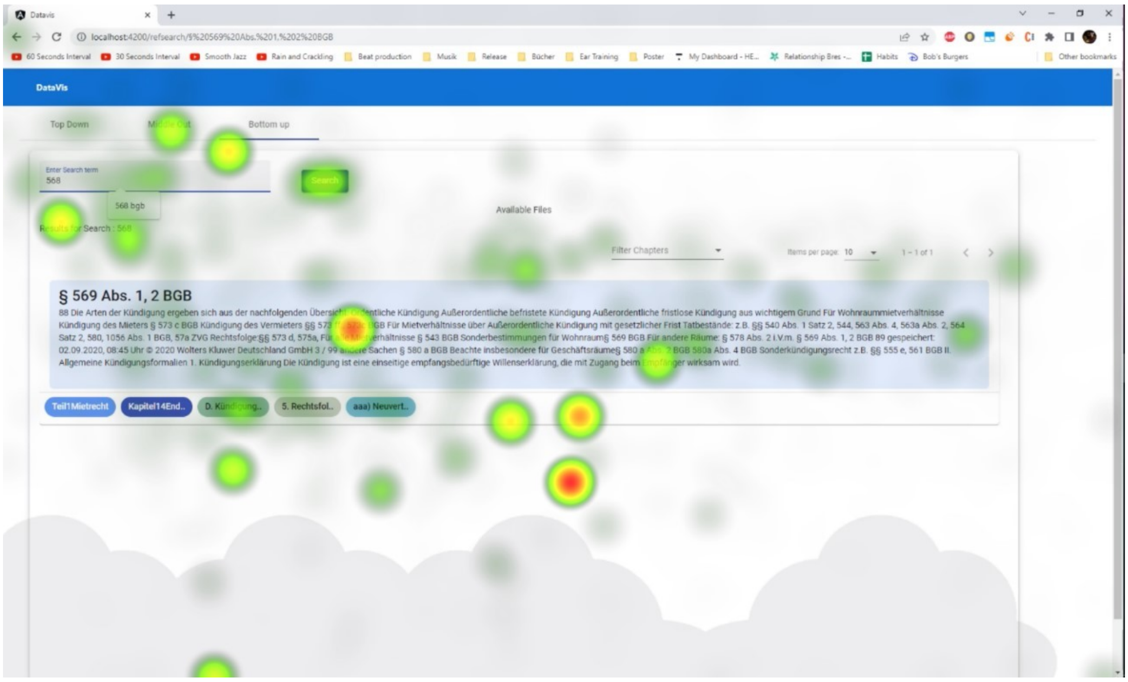

Figure S16. Heatmap from participants using the mouse in the 'Bottom-Up' approach
